# Tofacitinib ameliorates *Campylobacter-*induced intestinal pathology by suppressing IFNγ producing ILCs and T cells

**DOI:** 10.1016/j.mucimm.2025.06.010

**Published:** 2025-07-03

**Authors:** Anna A. Korchagina, Sergey A. Shein, Wayne T. Muraoka, Justin Nguyen, Qiangxing Chen, Anna A. Tumanova, Austin W. Todd, Carlos E. Rivera, Rita Tamayo, Paolo Casali, Ekaterina Koroleva, Alexei V. Tumanov

**Affiliations:** a Department of Microbiology, Immunology and Molecular Genetics, University of Texas Health Science Center at San Antonio, 7703 Floyd Curl Dr., San Antonio, TX 78229, USA; b Department of Gastroenterology, The Second Xiangya Hospital, and Research Center of Digestive Disease, Central South University, Changsha, Hunan 410011, China; c Department of Microbiology and Immunology, University of North Carolina at Chapel Hill School of Medicine, Chapel Hill, NC, USA

**Keywords:** Tofacitinib, JAK/STAT, Infectious colitis, Innate lymphoid cells, *Campylobacter jejuni*

## Abstract

Patients with autoimmune diseases are more susceptible to foodborne infections, which can be exacerbated by immunosuppressive therapy. Tofacitinib, a JAK/STAT pathway inhibitor, was recently approved for the treatment of ulcerative colitis, yet its effects on the pathogenesis of intestinal infections remain unclear. Here, we examined the impact of oral tofacitinib treatment in a mouse model of *Campylobacter jejuni* (*C. jejuni*) infection. Our results show that early tofacitinib administration attenuates intestinal pathology without affecting bacterial colonization. Specifically, tofacitinib suppressed CXCL1, CXCL2, CCL2 chemokine expression by intestinal epithelial cells, limiting recruitment of monocytes and neutrophils to the gut. In addition, JAK/STAT inhibition reduced IFNγ-producing innate lymphoid cells (ILCs) and T cells in the gut. Furthermore, tofacitinib suppressed IFNγ production and ameliorated intestinal disease in humanized mice. Cell-fate mapping revealed that tofacitinib predominantly inhibited IFNγ production by NK1.1^−^ ILCs derived from NKp46^−^ progenitors and reduced NK1.1^−^ ILC proliferation without affecting ILC3 to ILC1 plasticity. Notably, tofacitinib ameliorated intestinal disease even in the absence of T cells. These findings suggest that tofacitinib alleviates *C. jejuni*-induced colitis by reducing proinflammatory cytokine production by monocytes/macrophages/epithelial cells and suppressing IFNγ secretion by ILCs and T cells, while preserving antibacterial defenses.

## Introduction

Immunosuppressive therapies which target proinflammatory cytokines have been extensively applied for the treatment of autoimmune and inflammatory diseases.^1,2^ The JAK/STAT pathway controls expression of numerous genes which promote inflammatory immune responses in various human diseases.^1,2^ Pharmacological targeting of the JAK/STAT pathway has been shown to be effective as an immunosuppressive therapy for patients with autoimmune diseases.^1,2^ Tofacitinib (CP-690550) is a small-molecule JAK inhibitor which primarily blocks JAK1, JAK3, and with less affinity JAK2 activation mediated by multiple cytokines.^2,3^ It was licensed for the treatment of rheumatoid arthritis and psoriatic arthritis.^1^ More recently, tofacitinib has been approved for the immunosuppressive therapy of ulcerative colitis (UC), one of the forms of inflammatory bowel disease (IBD).^4^ IBD pathogenesis is associated with excessive production of JAK-dependent cytokines, such as IFNγ, IL-6, IL-23.^2,5^ Since the JAK/STAT pathway controls IFNγ-mediated host protection, a major concern of using JAK inhibitors is the weakened immune response against viral and bacterial infections in immunocompromised patients.^2,6^ Although clinical trials showed modest risk of infections during tofacitinib treatment, recent studies reported that long-term suppression of the JAK/STAT pathway impairs mucosal immunity, leading to increased risk of opportunistic infections.^2,4,6,7^ However, the effects of tofacitinib on pathogenesis of enteric infections remain largely unknown.

The foodborne pathogen *Campylobacter jejuni* (*C. jejuni*) is one of the most common causes of bacterial human gastroenteritis in the US.^8^ While *C. jejuni* infection is mild and usually self-resolving in healthy individuals, serious and long-lasting disease can occur in immunocompromised patients, children, and older individuals.^9^ *C. jejuni* post-infection complications include Guillain-Barré syndrome, irritable bowel syndrome, and reactive arthritis.^9^ Moreover, the incidence of *C. jejuni* infection in UC patients correlates with worse clinical out-comes.^10^ IL-10 deficient mice represent a well-established model of intestinal inflammation and are widely used to study the pathogenesis of IBD.^11^ In mice with impaired IL-10R signaling, *C. jejuni* induces intestinal pathology similar to that in humans.^12^ *C. jejuni* infection in human patients and in mouse models leads to increased levels of JAK-dependent cytokines such as IFNγ and IL-6 along with JAK-independent cytokine IL-1β.^13-15^ IFNγ and IL-6 signal through JAK1/JAK2 receptor-associated kinases to activate STAT1 and STAT3, respectively.^2,16^ We recently identified the critical role of IFNγ-producing innate lymphoid cells (ILCs) in driving *C. jejuni*-induced colitis during the early stages of infection.^14^ The JAK/STAT pathway participates in the proliferation and homeostasis of ILCs.^17,18^ However, it remains unknown whether tofacitinib treatment affects ILCs-mediated immune response in the context of intestinal bacterial infection.

In the present study, we investigated the impact of tofacitinib treatment on infectious colitis caused by *C. jejuni*. We found that pharmacological inhibition of the JAK/STAT pathway ameliorated intestinal inflammation driven by IFNγ producing T cells and ILCs without affecting the antimicrobial response. We revealed that tofacitinib treatment inhibits NK1.1^−^ ILC1s, ex-ILC3s, and T cells locally in the colon but not in the mesenteric lymph node. Furthermore, tofacitinib treatment reduced *C. jejuni*-induced intestinal disease and IFNγ production by human immune cells in humanized mice, collectively suggesting that JAK/STAT pathway inhibition does not compromise protection against *C. jejuni*.

## Results

### Tofacitinib ameliorates C. jejuni-induced intestinal inflammation without compromising antibacterial responses

**Fig. 1.**
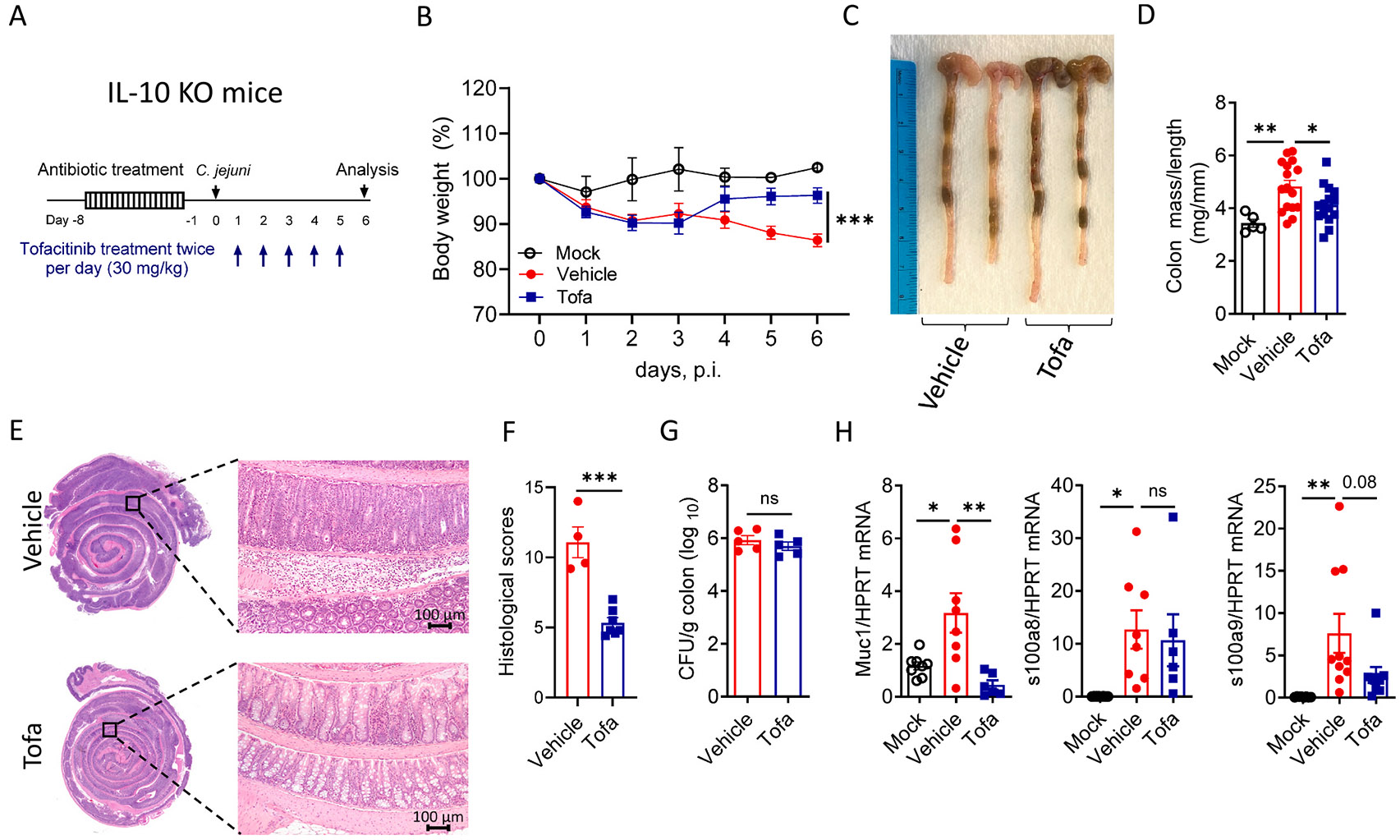
Tofacitinib ameliorates *C. jejuni*-induced intestinal inflammation without compromising bacterial colonization. (A) Schematic of the experimental design. (B) Body weight change (n=2–10). (C-D) Representative photographs and colon mass-to-length measurements (n=5–16). (E) Representative H&E staining of the colon. Left: representative panoramic colon images (x2 magnification), right: representative colon images (x10 magnification). Scale bar, 100 μm. (F) Histological scores (n=4–7). (G) Bacterial loads in the colon (n=5/group). (H) Expression of mucin 1 (Muc1), s100a8 and s100a9 in the colon (n=6–9). Each dot represents an individual mouse. (B, F-G) two-tailed unpaired *t*-test, (D, H) one-way ANOVA with Dunnett’s multiple comparisons test. Data shown as mean±SEM. ns- not significant, *p<0.05, **p<0.01, ***p<0.001.

To assess the impact of tofacitinib treatment on the sensitivity to intestinal infection induced by human enteric pathogen *C. jejuni*, we utilized IL-10 deficient mice (IL-10 KO), a well-established mouse model of chronic intestinal inflammation.^12,13^ IL-10 KO mice were treated with antibiotic cocktail for 7 days to promote bacterial colonization. One day after antibiotic withdrawal, mice were orally infected with *C. jejuni* and treated orally twice daily with 30 mg/kg of tofacitinib (Tofa) or vehicle control (0.5% methylcellulose/0.025% Tween 20) (Fig. 1A) By day 6 post-*C. jejuni* infection (pi), mice developed intestinal inflammation accompanied by body weight loss and thickening of the colon (Fig. 1B-D), consistent with previous studies.^14,19^ Furthermore, infected mice exhibited histopathological signs of colitis, including infiltration of immune cells in the colonic lamina propria and epithelial cell damage (Fig. 1E). Strikingly, tofacitinib-treated infected mice started to gain weight from day 4 pi (Fig. 1B), and demonstrated attenuated intestinal inflammation as evidenced by improved colon thickness and ameliorated intestinal histopathology (Fig. 1C-F). Tofacitinib-treated mice and control mice demonstrated comparable bacterial loads in the colon (Fig. 1G), suggesting that tofacitinib did not affect *C. jejuni* colonization.

To further define the effect of tofacitinib on antimicrobial response, we measured expression of mucin 1 (Muc1) and antimicrobial proteins S100a8/S100a9 which contribute to *C. jejuni* colonization.^20,21^ Expression of Muc1 and S100a8/S100a9 was upregulated following *C. jejuni* infection, consistent with previous studies.^20,21^ Oral tofacitinib treatment after infection had no effect on S100a8 expression while Muc1 expression was downregulated and S100a9 expression showed a trend to reduction (Fig. 1H). Thus, our data suggests that inhibition of the JAK/STAT pathway ameliorates intestinal inflammation without compromising bacterial colonization.

Given that JAK/STAT signaling is important for antimicrobial response, we next evaluated the impact of tofacitinib on early and late responses to *C. jejuni* infection. First, we treated infected mice with tofacitinib on days 1–5 pi and monitored them until day 9 (Fig. S1A). Stopping tofacitinib treatment at day 5 led to continued body weight loss in tofacitinib-treated mice, although this body weight loss was less pronounced compared to the vehicle-treated group (Fig. S1A). The tofacitinib group also showed a non-significant trend toward reduction of colon mass-to-length ratio (Fig. S1C). Second, to determine whether JAK/STAT signaling can ameliorate already established *C. jejuni*-induced intestinal disease, we treated mice with tofacitinib on days 5–8 pi (Fig. S1B). Intestinal inflammation was comparable between groups (Fig. S1B and C). Notably, tofacitinib did not alter *C. jejuni* bacterial loads (Fig. S1D). Collectively, these results suggest that tofacitinib is critical for ameliorating early *C. jejuni*-induced intestinal immunopathology.

To test the impact of tofacitinib treatment in a different enteric infection, we employed a mouse *Clostridioides difficile* infection (*C. difficile*) model.^22^ Antibiotic pretreated C57BL/6 mice were orally inoculated with 10^8^ CFU of *C. difficile* R20291 spores. Starting one day post-infection, mice orally received tofacitinib or vehicle control twice daily for 4 days (experimental design Fig. S1E). Tofacitinib-treated mice demonstrated faster recovery from body weight loss, improved gross colon pathology, and longer colons than the vehicle-treated group (Fig. S1F-H). These findings indicate that broad JAK/STAT inhibition alleviates intestinal inflammation caused by *C. difficile* infection.

### JAK/STAT inhibition reduces proinflammatory cytokine expression after C. jejuni infection

**Fig. 2.**
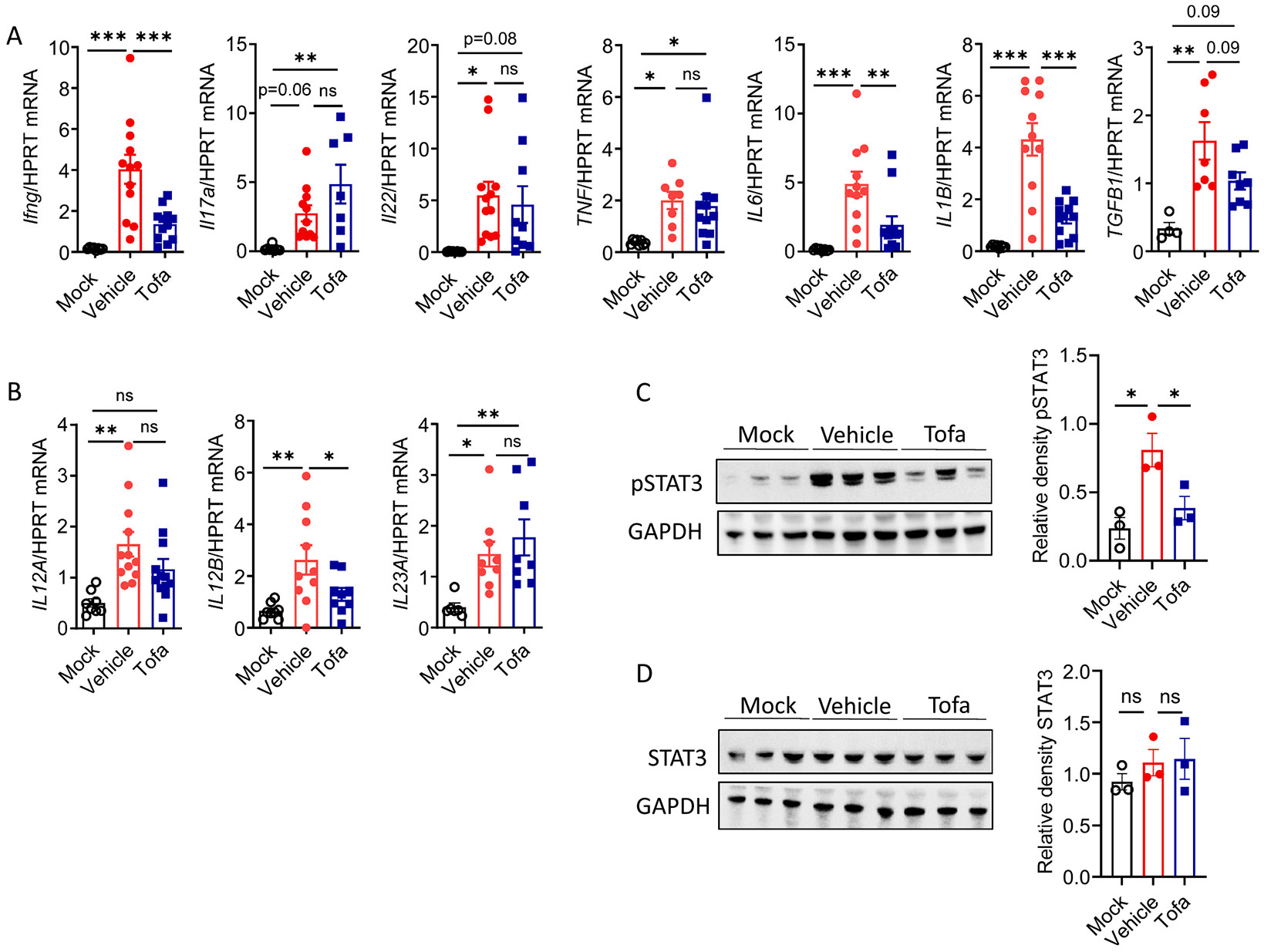
JAK/STAT inhibition decreases expression of proinflammatory cytokines in the colon. (A-B) Expression of cytokines was measured in the colon of mock- or *C. jejuni*- infected IL-10 KO mice treated with vehicle or Tofa at day 6 pi by real-time PCR (n=4–12). (C-D) STAT3 phosphorylation (pSTAT3) and total STAT3 levels in the colon was determined by Western blot analysis. (C) Representative blots of pSTAT3 and relative density of pSTAT3 to GAPDH (n=3/group). (D) Representative blots of STAT3 and relative density of STAT3 to GAPDH (n=3/group). Each symbol represents an individual mouse. (A-B) Data are pooled from three independent experiments. (C-D) Data is representative of one out of three independent experiments. Data shown as mean±SEM. ns- not significant, *p<0.05, **p<0.01, ***p<0.001, one-way ANOVA with Dunnett’s or with Tukey’s multiple comparisons test.

*C. jejuni* infection stimulates production of proinflammatory cytokines in the colon.^13-15^ We found increased expression of JAK-dependent cytokines such as IFNγ, IL-22, IL-6, IL-12, and IL-23, as well as cytokines that signal independently of the JAK pathway, including IL-17A, IL-1β, TNF, and TGFβ.^2,23^ in the colons of IL-10 KO mice at day 6 pi (Fig. 2A and B), in agreement with previous studies.^13-15^ Tofacitinib reduced expression of JAK-activating cytokines IFNγ (JAK1/2), IL-6 (JAK1/2), and IL-12p40 (JAK2/TYK2),^16^ whereas only one JAK-independent cytokine, IL-1β, was reduced in tofacitinib-treated mice (Fig. 2A and B). The expression of IL-17a, IL-22, TNF, TGFβ, and IL-23p19 did not change (Fig. 2A and B).

Since the JAK/STAT pathway is an important regulator of intestinal epithelial cell (IEC) homeostasis, we further assessed the impact of global JAK/STAT suppression on IECs during the early stage of *C. jejuni* infection, when intestinal inflammation was comparable between groups (Fig. S2A and B). To test this, we isolated colonic IECs on day 3 pi. Flow cytometry analysis confirmed that majority of isolated cells in the epithelial fraction were CD45^−^ EpCAM^+^ cells (Fig. S2D). The number of Ki-67^+^ IECs and Ki-67 expression in the colon on day 3 pi were comparable between tofacitinib and vehicle-treated groups (Fig. S2E-F), suggesting that proliferation of IECs was not affected by tofacitinib treatment. Since STAT3 is required for the maintenance of the epithelial barrier,^24^ we evaluated STAT3 activity in epithelial cells on day 3 pi. Although pSTAT3 was not affected by tofacitinib treatment in IECs, there was a trend in reduction of pSTAT3 in colon lamina propria (cLP) (Fig. S2G-H). By day 6 pi, tofacitinib markedly inhibited STAT3 activation without altering total STAT3 levels in the colon (Fig. 2C and D). These data suggest that pan-JAK/STAT inhibition predominantly affects STAT3 activation in cLP compared to IECs during *C. jejuni* infection.

To assess the direct effect of tofacitinib on IECs during *C. jejuni* infection, we treated mouse colonic epithelial cells CMT-93 with live *C. jejuni* for 4 h and then added tofacitinib for 16 h (Fig. S2I). *C. jejuni* infection increased the expression of IL-6 and TNF as well as chemokines CCL2, CXCL1, and CXCL2 (Fig. S2J and K). Tofacitinib reduced expression of these chemokines and IL-6 without affecting TNF expression (Fig. S2J and K). Moreover, JAK/STAT inhibition suppressed STAT3 activity (Fig. S2L). These data suggest that tofacitinib can directly suppress STAT3 activation in IECs *in vitro*.

### JAK/STAT inhibition reduces accumulation of neutrophils and monocytes/macrophages in the colon

**Fig. 3.**
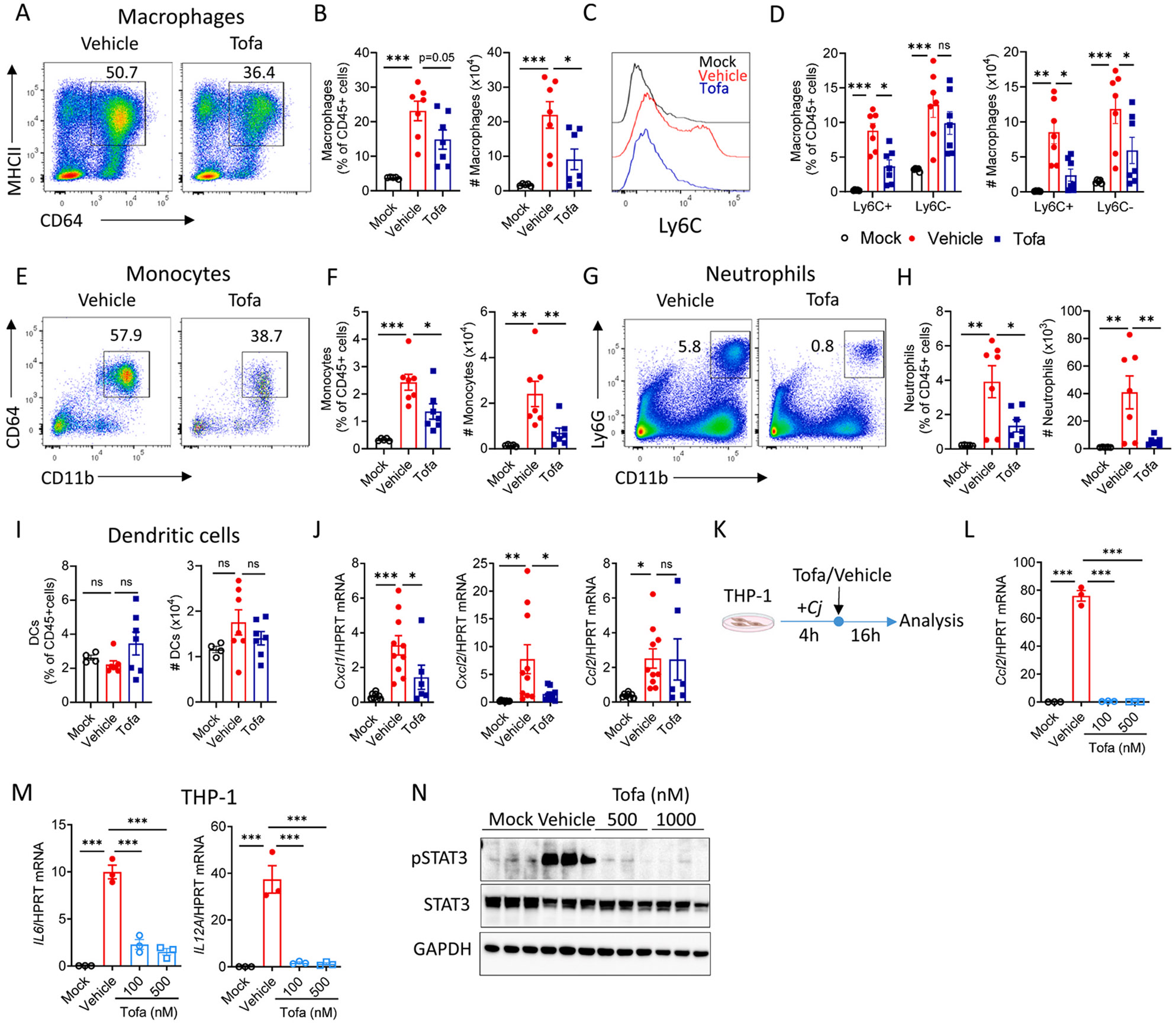
Tofacitinib reduces accumulation of macrophages, monocytes and neutrophils in the colon. IL-10 KO mice were treated as described on Fig. 1A. Cells were purified from colon lamina propria on day 6 pi (A) Representative flow cytometry plots of macrophages (Ly6G^−^ CD64^+^MHCII^+^CD11b^+^). (B) Frequency and cell numbers of macrophages (n=5–7). (C) Representative histogram of Ly6C expression on macrophages. (D) Frequency and cell numbers of Ly6C^+^ and Ly6C^−^ macrophages (n=5–7). (E) Representative flow cytometry plots and (F) frequency and cell numbers of monocytes (Ly6G^−^ MHCII^−^ CD64^+^CD11b^+^) (n=5–7). (G) Representative flow cytometry plots and (H) frequency and cell numbers of neutrophils (Ly6G^+^, n=5–7). (I) Total cell numbers and frequency of dendritic cells (n=4–7). (J) Expression of chemokines in the colon measured by real-time PCR (n=6–10). Data were normalized to *hprt* expression. (K-N) THP-1 cells were mock-treated or *C. jejuni*-infected for 4 h, then given tofacitinib (Tofa) or vehicle and incubated 16 h before analysis. (K) Schematic of experimental design. Expression of (L) CCL2 and (M) proinflammatory cytokines (n=3). (N) Representative blots of pSTAT3 and STAT3 (n=3/group) following *C. jejuni* infection and treatment with tofacitinib. (A-J) Data are pooled from two independent experiments. (L-N) Data is representative of one out of two independent experiments. Each symbol represents (A-J) an individual mouse or (L-N) biological replicate. Data shown as mean ± SEM. ns- not significant, *p<0.05, **p<0.01, ***p<0.001. (A-B, E-J, L-M) one-way ANOVA with Dunnett’s multiple comparisons test, (D) two-way ANOVA with Tukey’s multiple comparisons test.

*C. jejuni*-driven pathology is associated with infiltration of immune cells into the colon.^13^ Macrophages, monocytes, and neutrophils accumulate in the colon at 6 days pi (Fig. 3A-I, gating strategy is shown in Fig. S3). Previous studies demonstrated that monocytes, macrophages and dendritic cells produce proinflammatory cytokines upon *C. jejuni* infection.^25-27^ Considering reduced proinflammatory cytokine production after tofacitinib treatment (Fig. 2A and B), we hypothesized that JAK/STAT inhibition can affect accumulation of myeloid cells in the colon. Consistent with our hypothesis, we detected fewer monocytes (Ly6G^−^ MHCII^−^ CD11b^+^CD64^+^Ly6C^+^), macrophages (Ly6G^−^ MHCII^+^ CD11b^+^CD64^+^) and neutrophils (Ly6G^+^CD11b^+^) in the colon after tofacitinib treatment compared to vehicle-treated controls (Fig. 3A-H).

Ly6C^hi^ monocytes migrate from the blood to the inflamed intestine where they differentiate into macrophages through an intermediate stage.^28,29^ Previous studies described Ly6C^+^MHCII^+^ cells as an intermediate stage between monocytes (Ly6C^+^MHCII^−^) and resident macrophages (Ly6C^−^ MHCII^+^).^29^ We found that *C. jejuni* infection drives accumulation of both Ly6C^+^ and Ly6C^−^ macrophages in the colon (Fig. 3C and D). Tofacitinib prevented accumulation of Ly6C^+^ macrophages in infected colons, while the frequency of Ly6C^−^ macrophages did not change (Fig. 3D). In contrast to macrophages, dendritic cell numbers were comparable between all groups (Fig. 3I). Expression of neutrophil- and monocyte-recruiting chemokines CXCL1, CXCL2, and CCL2 was increased in the colon after *C. jejuni* infection (Fig. 3J). Consistent with reduction of intestinal neutrophils after JAK/STAT inhibition, CXCL1 and CXCL2 expression was reduced in the colon after tofacitinib treatment (Fig. 3J). Together, these data suggest that tofacitinib reduces the influx of neutrophils, monocytes, and macrophages to the colon during *C. jejuni* infection.

Since neutrophils and monocytes are the first cells to enter the colon from the blood during infection, we analyzed circulating cells in the blood on day 3 pi (Fig. S4A). Tofacitinib treatment had no effect on neutrophil recruitment either in blood or in cLP at day 3 post-*C. jejuni* infection (Fig. S4B and C). In contrast, we found a significant reduction in monocytes in the blood of tofacitinib-treated mice, resulting in fewer monocytes and macrophages in the cLP (Fig. S4B and C). We did not find differences in cytokine and chemokine expression between the groups in the colon tissue on day 3 pi (data not shown). To determine if tofacitinib treatment directly affects cytokine and chemokine production by monocytes during *C. jejuni* infection, we treated human monocytic leukemia cell line THP-1 with live *C. jejuni* and tofacitinib (Fig. 3K). *C. jejuni* infection increased expression of CCL2, IL-6, and IL-12p35 whereas tofacitinib treatment reduced their expression (Fig. 3L and M). Consistent with reduced IL-6 expression, tofacitinib treatment abrogated *C. jejuni*-induced STAT3 activation in THP-1 cells (Fig. 3N). These data suggest that tofacitinib can directly inhibit proinflammatory cytokines production by monocytes.

### Tofacitinib inhibits IFNγ production by ILC1s and T cells

**Fig. 4.**
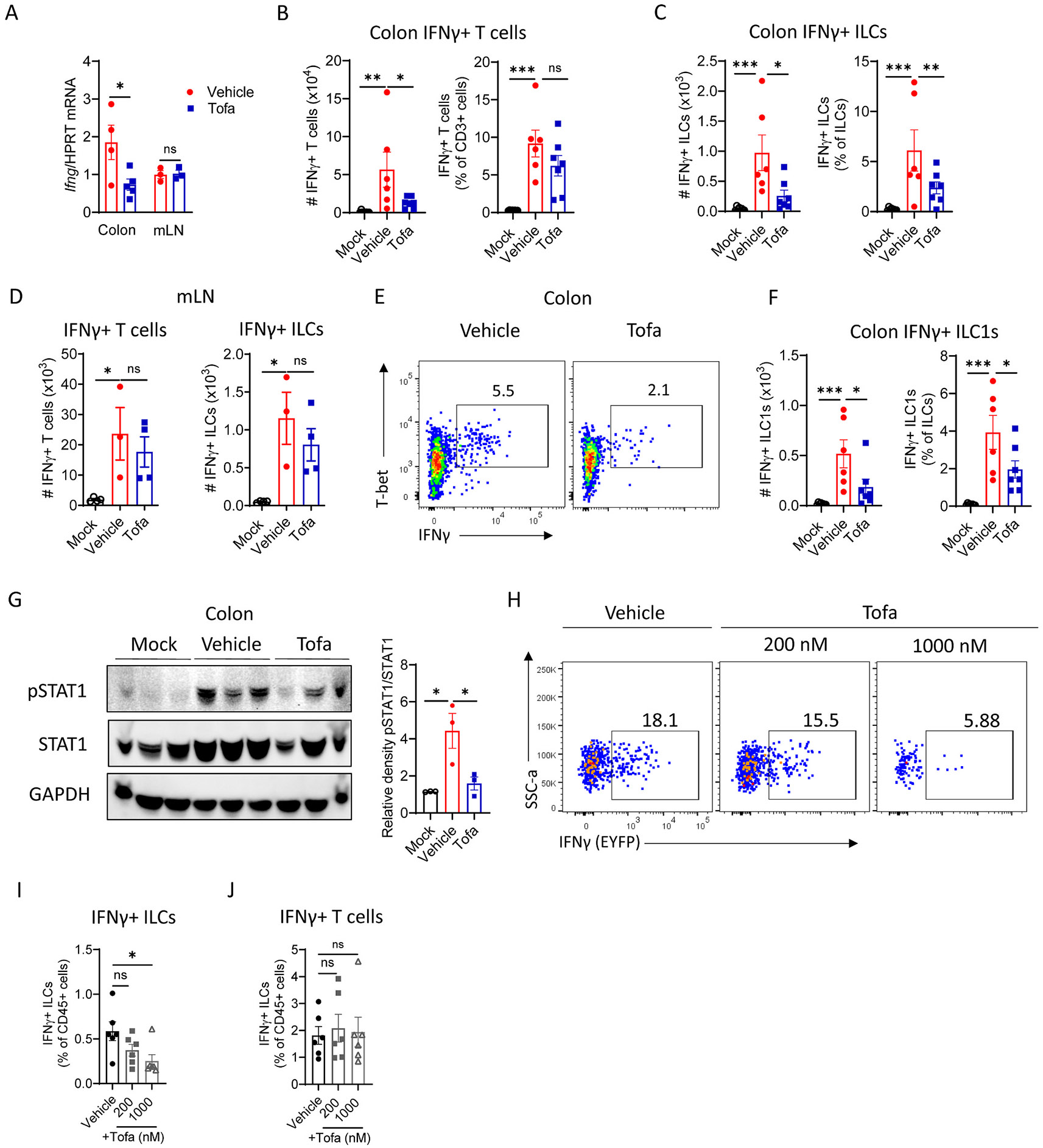
Tofacitinib inhibits IFNγ production by ILC1s and T cells. (A) IFNγ expression was measured by real-time PCR in the colon (n=4) and mesenteric lymph node (mLN) (n=3) of *C. jejuni* infected vehicle- or tofa-treated IL-10 KO mice on day 6 pi. Data were normalized to *hprt* expression. (B-F) Cells were isolated from the colon lamina propria of IL-10 KO mice at day 6 pi For IFNγ assessment cells were stimulated with PMA and ionomycin in the presence of Brefeldin A (n=6–7). (B) Total cell numbers and frequency of IFNγ^+^ T cells (CD3^+^). (C) Total cell numbers and frequency of IFNγ^+^ ILCs. (D) Cell numbers of IFNγ-producing T cells and ILCs in mLN (n=3–4). (E) Representative flow cytometry plots of IFNγ-producing ILC1s (CD3^−^ Lin^−^ Thy1^+^Eomes^−^ T-bet^+^) in the colon. Flow plots show percentage of IFNγ^+^ ILC1s among ILCs. (F) Total cell numbers and frequency of IFNγ^+^ ILC1s (n=6–7). (G) Representative blots of pSTAT1/STAT1 and relative density of pSTAT1 to STAT1 in the whole colon on day 6 pi. (H-J) IFNγ-reporter mice were treated with aIL-10R blocking antibodies prior to infection with *C. jejuni* as described in Materials and Methods. Colon lamina propria cells were isolated from infected mice and stimulated with PMA and ionomycin in the presence of vehicle or 200 nM or 1000 nM of tofa for 16 h. (H) Representative flow cytometry plots of IFNγ-producing ILCs (CD45^+^CD3^−^ Lin^−^ Thy1^+^). (I) Frequency of IFNγ^+^ ILCs after stimulation *ex vivo* in the presence of tofa (n=6). (J) Frequency of IFNγ^+^ T cells (CD45^+^B220^−^ CD3^+^) after stimulation *ex vivo* in the presence of tofa (n=6); Lineage- (Lin^−^): B220^−^ CD11c^−^ Ter119^−^ Gr1^−^ CD5^−^. Each symbol represents an individual mouse. Data are pooled from two independent experiments. Data shown as mean±SEM. ns- not significant, *p<0.05, **p<0.01, ***p<0.001. (A) two-tailed unpaired *t*-test, (B-G) one-way ANOVA with Dunnett’s or (I-J) with Tukey’s multiple comparisons test.

*C. jejuni* stimulates production of IFNγ, which drives intestinal pathology.^13,14^ Notably, oral tofacitinib treatment of *C. jejuni*-infected mice reduced IFNγ expression in the colon but not in the mesenteric lymph node (mLN) (Fig. 4A), suggesting that oral tofacitinib treatment restrains IFNγ production by immune cells locally in the colon without affecting other organs.

To determine which IFNγ-producing cells were affected by tofacitinib treatment, we next analyzed cells isolated from cLP and mLN by flow cytometry (gating strategy is shown in Fig. S5A). Tofacitinib administration reduced the number of IFNγ-producing T cells (CD3^+^) and ILCs (CD3^−^ Lin^−^ Thy1^+^Eomes^−^) in the cLP but not in the mLN (Fig. 4B-D). In contrast, IFNγ production by NK cells in the colon was not affected by tofacitinib treatment (Fig. S5B). Additionally, the total number of T cells in the colon was reduced by tofacitinib treatment (Fig. S5C), whereas the number of ILCs and NK cells was not affected (Fig. S5D and E). Notably, tofacitinib treatment did not change the number of T cells and ILCs in mLN (Fig. S5F). Because the vast majority of IFNγ–producing ILCs are KLRG1^−^ and T-bet^+^ (ILC1),^14^ we focused our analyses on this population, although a small subset of KLRG1^+^T-bet^+^ cells also produces IFNγ (Fig. S5I). Flow cytometry analysis revealed reduced number of IFNγ^+^ ILC1s (Fig. 4E and F) in the colon of tofacitinib-treated mice, whereas reduction of IFNγ-producing ILC3s was not significant (Fig. S5G and H). Moreover, *C. jejuni* infection induced STAT1 phosphorylation in the colon (Fig. 4G). STAT1 is activated predominantly by IFNγ through JAK1/JAK2-dependent signalling.^2,16^ Consistent with reduced IFNγ levels we found reduced STAT1 activation in the colon of tofacitinib-treated mice (Fig. 4G).

To confirm that tofacitinib inhibits IFNγ production by ILCs *in vitro*, we isolated cells from *C. jejuni*-infected IFNγ-EYFP reporter mice and stimulated them with PMA and ionomycin with or without tofacitinib. After 16 h, IFNγ production in ILCs (CD45^+^CD3^−^ B220^−^ Lin^−^ Thy1^+^) was significantly lower in the tofacitinib-treated group compared to the vehicle group (Fig. 4H and I). In contrast, T cells produced similar levels of IFNγ in both tofacitinib- and vehicle-treated groups (Fig. 4J), indicating that *ex vivo* tofacitinib treatment specifically inhibited IFNγ production by ILCs but not by T cells. Collectively, these results indicate that oral tofacitinib treatment inhibits IFNγ production by both ILC1s and T cells locally in the colon during *C. jejuni* infection without affecting systemic IFNγ-driven immune response.

### Tofacitinib suppresses C. jejuni-induced IFNγ production in mice with human immune system

**Fig. 5.**
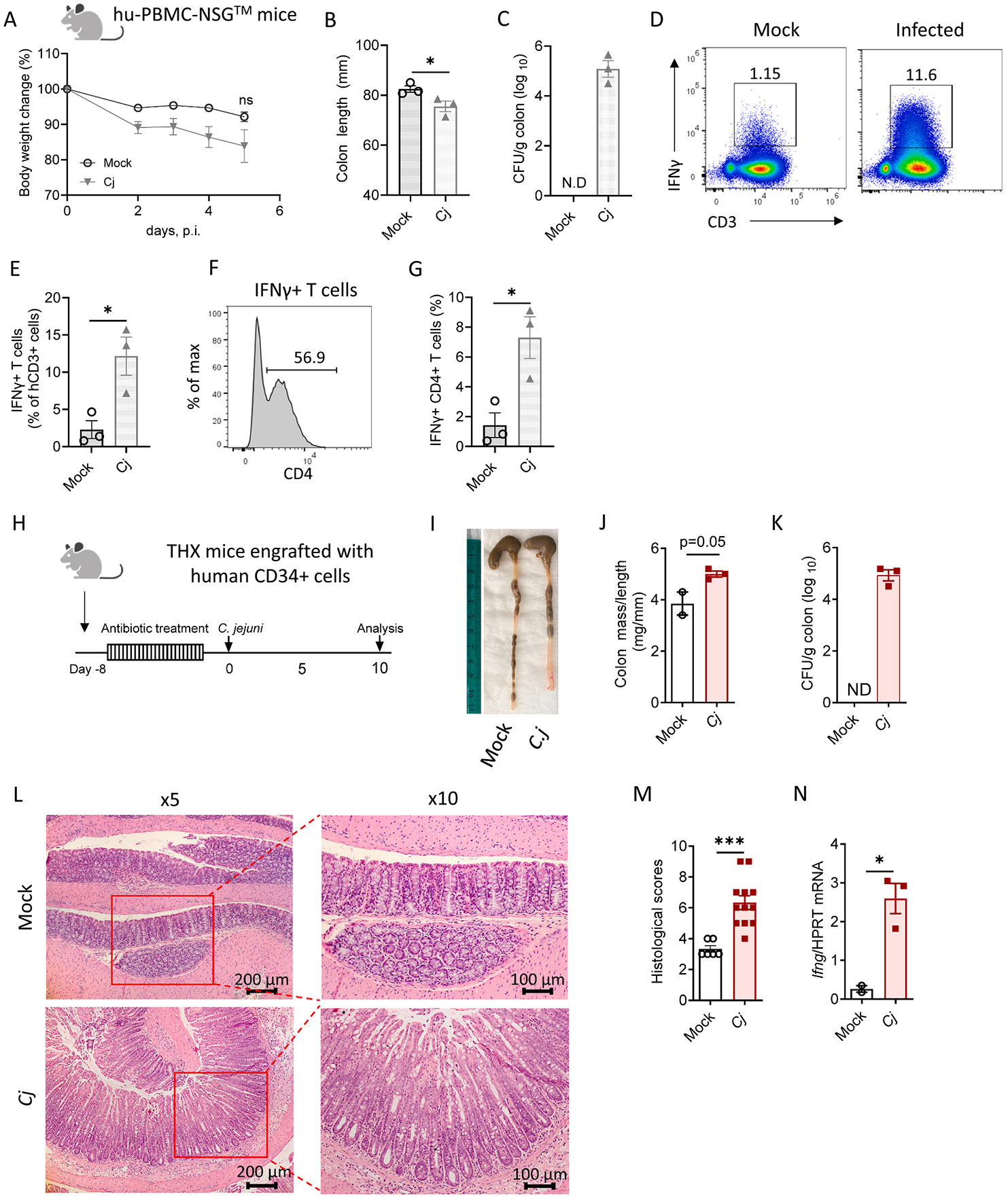
*C. jejuni* infection promotes intestinal inflammation and IFNγ production in mice with human immune system. (A-G) NSG mice reconstituted with human PBMCs were treated as described in Materials and Methods and mock-infected (Mock) or orally infected with *C. jejuni* (*Cj*). (A) body weight loss, (B) colon length, (C) bacterial loads in the colon were analyzed at day 5 pi. (D-G) Colon lamina propria cells were stimulated with PMA and ionomycin in the presence of Brefeldin A. Cells were gated on live, hCD45^+^ cells. (D) Representative flow plots and (E) frequency of human IFNγ-producing T cells (hCD3^+^). (F) Representative histogram of CD4 expression on IFNγ^+^ T cells. (G) Frequency of IFNγ-producing human CD4^+^ T cells. (H-N) Analysis of *C. jejuni*-induced colitis in THX humanized mice. NBSGW (NOD. Cg-*Kit*^*W*–*41J*^ *Tyr* ^+^ *Prkdc*^*scid*^ *Il2rg*^*tm1Wjl*^/ThomJ) mice were engrafted with human cord blood CD34^+^ cells followed by 17β-estradiol conditioning. (I) Representative photographs and (J) colon mass-to-length measurements (n=2–3). (K) *C. jejuni* colonization in the colon (n=3). (L) Representative H&E images of the colon. (M) Histological scores. (N) IFNγ expression in the colon was measured by real-time PCR (n=2–3). Data were normalized to human hprt expression. Each symbol represents an individual mouse. Data is representative of one out of two experiments with similar results. Data shown as mean±SEM. ns- not significant, *p<0.05, two-tailed unpaired *t*-test.

To better understand how human immune cells respond to tofacitinib treatment in the context of foodborne pathogen infection, we utilized mice with a human immune system. NSG (NOD.Cg-*Prkdc*^*scid*^*Il2rg*^*tm1Wjl*^/SzJ) mice reconstituted with human peripheral blood mononuclear cells (hu-PBMC-NSG) were orally inoculated with *C. jejuni* and analyzed on day 5 pi (Fig. 5A-G). Notably, *C. jejuni* successfully colonized the large intestine of hu-PBMC-NSG mice (Fig. 5C). Although mock-infected hu-PBMC-NSG mice also demonstrated body weight loss, potentially due to the development of acute graft versus host disease (GvHD),^30,31^ *C. jejuni*–infected hu-PBMC-NSG mice exhibited substantial weight loss and significant colon shortening, compared to mock infected mice (Fig. 5A and B). Additionally, flow cytometry analysis of human T cells showed increased IFNγ production following *C. jejuni* infection (Fig. 5D and E). Consistent with previous data,^13,26^ *C. jejuni* induced IFNγ production by CD4^+^ T cells (Fig. 5F and G). Histological analysis of the colon revealed significant damage in both mock and *Cj* groups (data not shown), suggesting that acute GvHD in hu-PBMC-NSG mice complicates the separation of *C. jejuni* and GvHD-induced pathology.

To overcome GvHD development in hu-PBMC-NSG mice, we used improved NOD.Cg-*Kit*^*W*–41*J*^ *Tyr* ^+^ *Prkdc*^*scid*^ *Il2rg*^*tm1Wjl*^/ThomJ 17β-estradiol-conditioned humanized mice (THX) that have higher engraftment rate without radiation, longer lifespan, and are less prone to developing acute GvHD.^32^ We assessed intestinal pathology on day 10 pi in THX mice compared to uninfected controls (Fig. 5H and M). Consistent with our findings in hu-PBMC-NSG mice, *C. jejuni* successfully colonized the intestine of THX mice (Fig. 5K). While there was no significant difference in body weight loss (data not shown), we detected increase in the colon mass-to-length ratio after *C. jejuni* infection (Fig. 5I and J). Histological analysis showed moderate cells infiltration in the cLP with some areas extending into submucosa (Fig. 5L and M). This correlated with increased expression of human IFNγ in the intestine after infection (Fig. 5N). Taken together, these data suggest that *C. jejuni* infection causes intestinal pathology in humanized mice, accompanied by increased IFNγ production.

**Fig. 6.**
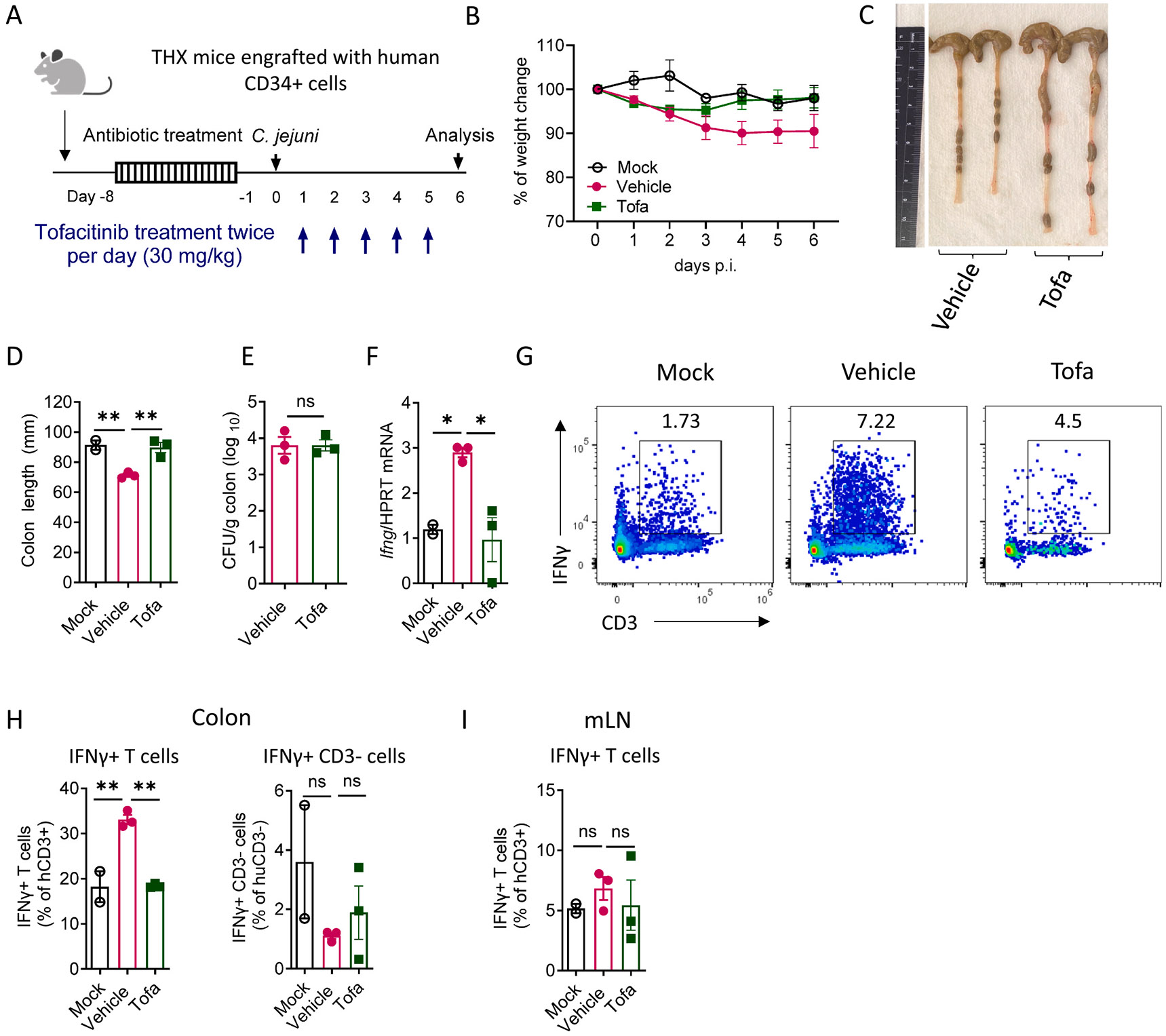
Tofacitinib suppresses *C. jejuni-*induced IFNγ production in mice with human immune system. (A) Experimental design of *C. jejuni*-induced colitis in THX humanized mice. THX mice were mock-infected (mock) or orally inoculated with *C. jejuni*. Infected mice were treated with either vehicle or 30 mg/kg tofacitinib by oral gavage twice daily after 1 day pi. Intestinal pathology was analyzed on day 6 pi. (B) Changes in body weight (n=3/group). (C-D) Representative photographs and colon length measurements (n=2–3). (E) *C. jejuni* titers in the colon (n=3/group). (F) IFNγ expression in the colon (n=2–3). (G) Representative flow cytometry plots and (H) frequency of live, human IFNγ^+^ T cells (CD3^+^) and CD3^−^ cells in the colon. (I) Frequency of human IFNγ^+^ T cells (CD3^+^) in the mesenteric lymph node (mLN). Each symbol represents an individual mouse. Data is representative of one out of two independent experiments. Data shown as mean±SEM. ns- not significant, *p<0.05, **p<0.01 (B, E) two-tailed unpaired *t*-test or (D, F-I) one-way ANOVA with Dunnett’s.

Next, we assessed whether tofacitinib treatment alleviates *C. jejuni*-driven IFNγ induction in the intestine (Fig. 6A-I). We found that on day 6 pi infected THX mice had shorter colons than mock controls, indicating ongoing intestinal inflammation caused by *C. jejuni* infection (Fig. 6C and D), with no significant difference in body weight loss (Fig. 6B). Vehicle-treated *C. jejuni* infected THX mice displayed higher IFNγ expression in the colon compared to mock-infected mice (Fig. 6F), consistent with our data in IL-10 KO mice (Fig. 2A). Tofacitinib treatment reduced colon shortening and decreased IFNγ expression (Fig. 6C, D and F), while bacterial loads were similar across groups (Fig. 6E). These data suggest that tofacitinib treatment ameliorates intestinal inflammation without affecting *C. jejuni* colonization. Consistently, tofacitinib treatment reduced IFNγ expression as well as frequency of human IFNγ^+^ T cells after *C. jejuni* infection (Fig. 6G and H).

Notably, the reduction of IFNγ was observed only in the colon but not in mLN (Fig. 6I), in agreement with data obtained in IL-10 KO mice (Fig. 4D). Interestingly, tofacitinib treatment did not change IFNγ production in human CD3^−^ cells (Fig. 6H), potentially due to variability of hCD45^+^CD3^−^ cell reconstitution in the intestine of humanized mice. Together, these data suggest that tofacitinib suppresses *C. jejuni*-induced intestinal IFNγ^+^ production predominantly by human T cells in humanized mice with functional IL-10 signaling.

### Tofacitinib inhibits IFNγ production by ILCs derived from NKp46^−^ progenitor cells

**Fig. 7.**
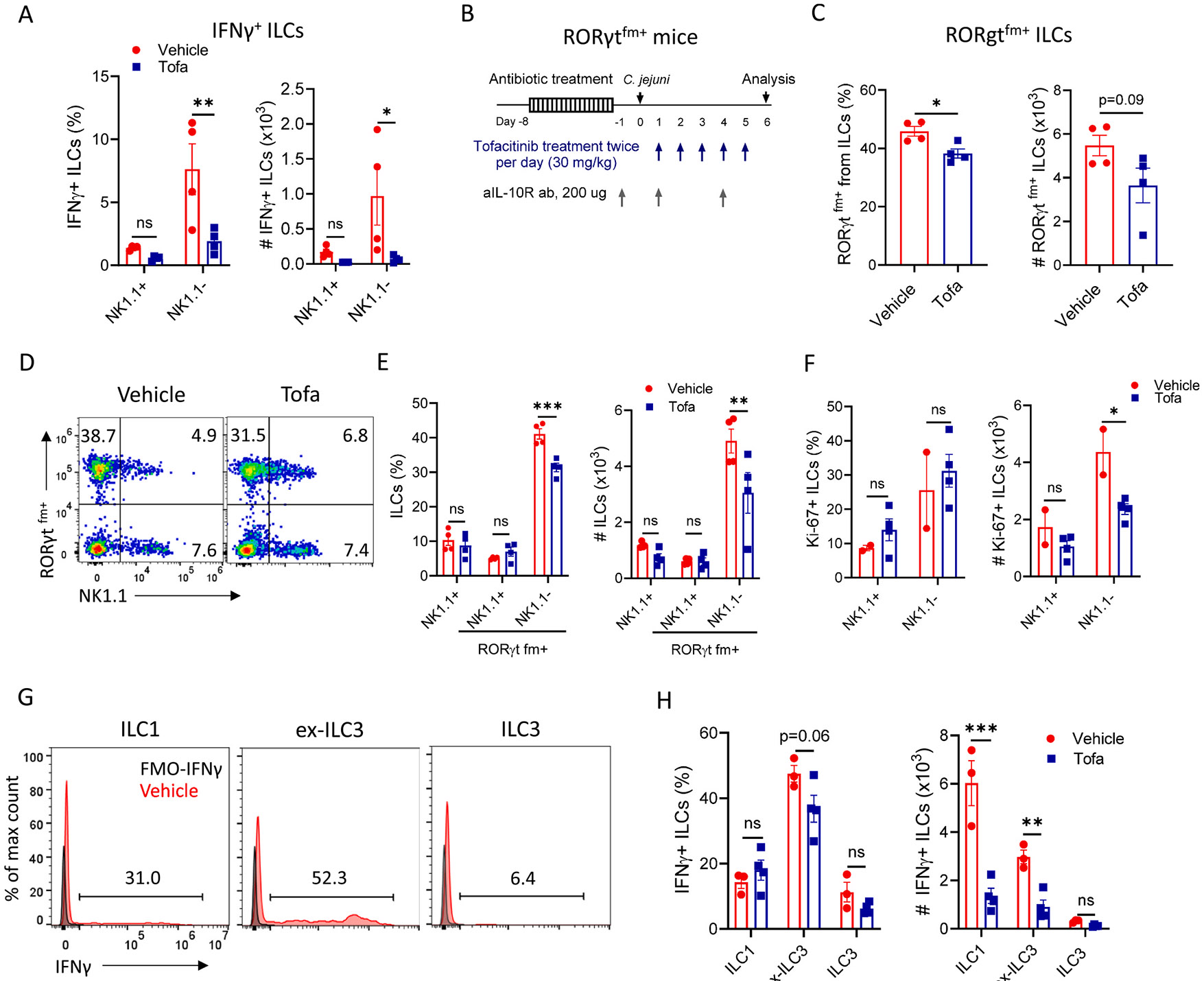
Tofacitinib inhibits IFNγ production by ILCs derived from NCR^−^ progenitor cells. Cells were isolated from the colon of *C. jejuni* infected vehicle- or tofa-treated mice on day 6 pi. (A) Frequency and total cell numbers of IFNγ-producing NK1.1^+^ and NK1.1^−^ ILCs (n=3–4) in *C. jejuni* infected IL-10 KO mice. (B-F) RORγt^fm+^ mice were treated as shown in (B). To render mice susceptible to *C. jejuni*-induced colitis, mice were treated with 200 μg of IL-10Rα blocking mAb (clone 1B1.3A from BioXCell, i.p.) on days −1, 1 and 4 pi. (C) Frequency and total cell numbers of RORγt^fm+^ ILCs (n=4); (D) Representative flow cytometry plots of NK1.1^+^ and NK1.1^−^ RORγt^fm+^ ILCs (CD3^−^ Lin^−^ Thy1^+^); Flow plots show percentage of NK1.1^+^ and NK1.1^−^ RORγt^fm+^ cells among ILCs. (E) Frequency and total cell numbers of NK1.1^+^ and NK1.1^−^ RORγt^fm+^ ILCs (n=4); (F) Frequency and total cell numbers of Ki-67^+^ ILCs in the cLP (n=2–4). (G) Representative histograms of IFNγ production in ILC1 (T-bet^+^RORγt^fm−^), ex-ILC3 (T-bet^+^RORγt^−^ RORγt^fm+^) and ILC3 (T-bet^−^ RORγt^+^RORγt^fm+^). (H) Frequency and total cell numbers of IFNγ-positive cells in indicated ILC subsets (n=3–4). Lineage: B220, CD11c, Ter119, Gr1, CD5. Each symbol represents an individual mouse. Data is representative of one out of three independent experiments. Data shown as mean±SEM. ns- not significant, *p<0.05, **p<0.01, ***p<0.001. (A, F) one-way ANOVA with Tukey’s multiple comparisons test, (C) two-tailed unpaired *t*-test, (D) two-way ANOVA with Sidak’s multiple comparisons test.

Mucosal NK cells and ILC1s co-express natural cytotoxicity receptors (NCRs), including NKp46 and NK1.1.^33^ Although both NK1.1^+^ and NK1.1^−^ ILCs produce IFNγ following *C. jejuni* infection, NK1.1^−^ ILCs are the predominant source of IFNγ (Fig. 7A). We found that tofacitinib treatment reduced IFNγ production by NK1.1^−^ ILCs and NKp46^−^ ILCs but not by NK1.1^+^ and NKp46^+^ ILCs (Fig. 7A, and Fig. S6A). These results suggest that JAK/STAT inhibition mainly affects IFNγ production by ILCs lacking NK1.1 and NKp46 expression.

Several studies showed that NKp46^−^ ILCs can lose the expression of NKp46 during development or under changing environmental conditions.^33,34^ To determine the developmental origin of NKp46^−^ IFNγ producing ILCs during *C. jejuni* infection, we employed NKp46 cell fate mapping approach. Therefore, we generated cell-fate NKp46^fm^ mice by crossing NKp46-Cre mice^35^ with Rosa26-TdTomato reporter mice.^36^ Analysis of intestinal ILCs from *C. jejuni*-infected NKp46^fm+^ mice on day 10 pi revealed that the majority of IFNγ-producing ILCs does not have a history of NKp46 expression (Fig. S6B-E). Thus, these results suggest that tofacitinib inhibits IFNγ production by colonic ILCs derived from NKp46^−^ progenitor cells.

### Tofacitinib inhibits proliferation of NK1.1^−^ ILCs without affecting ILC3 to ILC1 plasticity

Inflammatory conditions can induce phenotypical and functional plasticity of ILCs.^34,37^ A previous study using RORγt cell-fate mapping experiments suggested that *C. jejuni* infection drives ILC3 to ILC1 plasticity, and therefore IFNγ-producing ILCs are ex-ILC3s.^14^ Although STAT pathways can induce the production of cytokines required for the maintenance of ILC identity,^38^ it is unknown whether JAK/STAT inhibition can affect ILC plasticity. To test this hypothesis, we generated cell-fate RORγt^fm+^ mice by crossing RORγt-Cre mice^39^ with EYFP-reporter mice^36^ (Fig. 7B). We found that treatment with tofacitinib reduced the RORγt^fm+^ ILCs in the colon compared to vehicle-treated mice on day 6 pi (Fig. 7C). Further analysis revealed that tofacitinib treatment specifically reduced NK1.1^−^ RORγt^fm+^ ILCs but did not affect NK1.1^+^RORγt^fm+^ or NK1.1^+^RORγt^fm−^ ILCs (Fig. 7D and E). The population of T-bet^+^ ILCs includes both ILC1s and ex-ILC3s which lost the expression of RORγt. Because tofacitinib treatment inhibited IFNγ production by ILC1s (Fig. 4F), we next sought to characterize IFNγ production by ILC1s and ex-ILC3s in cLP. We defined ILC1s as T-bet^+^RORγt^fm−^ cells, ILC3s as RORγt^+^RORγt^fm+^ and ex-ILC3s as RORγt^−^ RORγt^fm+^ cells. Our analysis revealed that both ILC1s and ex-ILC3s produce IFNγ after *C. jejuni* infection (Fig. 7G and H). Moreover, tofacitinib treatment reduced the number of both IFNγ-producing ILC1s and IFNγ-producing ex-ILC3s in the colon (Fig. 7H). These results demonstrate that tofacitinib treatment inhibits IFNγ-producing ILC1s and ex-ILC3s in the colon in response to *C. jejuni* infection.

Given that tofacitinib can inhibit ILC proliferation,^17,18^ we assessed whether the reduction of NK1.1^−^ RORγt^fm+^ ILCs following tofacitinib treatment was due to decreased proliferation. We found less proliferating Ki-67^+^NK1.1^−^ ILCs in the cLP of tofacitinib-treated mice compared to vehicle-treated mice, whereas the number of NK1.1^+^ ILCs proliferating cells was not affected by tofacitinib treatment (Fig. 7F). Together, these data suggest that tofacitinib treatment predominantly inhibits proliferation of NK1.1^−^ ILCs without altering their plasticity.

### Tofacitinib ameliorates C. jejuni-induced intestinal pathology in T cell-deficient mice

**Fig. 8.**
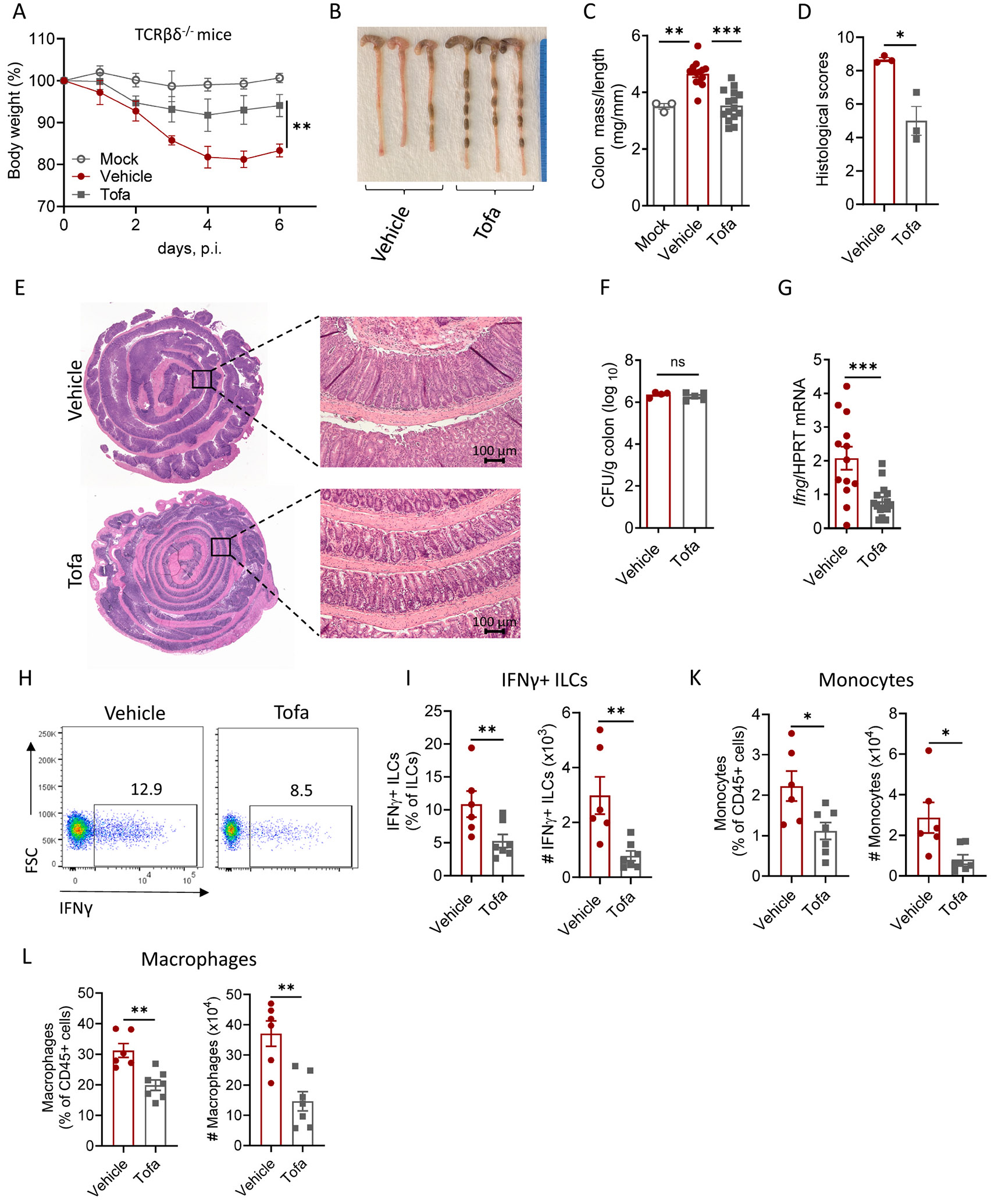
Tofacitinib ameliorates *C. jejuni*-induced intestinal inflammation in T cell-deficient mice by suppressing IFNγ production by ILCs. TCRβδ^−/−^ mice were treated according to the schematic on Fig. 7B. (A) Changes in body weight (n=7), (B-C) representative photographs and colon mass-to-length measurements (n=13–15), (D) Histological scores (n=3), (E) representative H&E staining of colon. Left - representative panoramic colon images (x2 magnification), right – representative colon images (x10 magnification). Scale bar, 100 μm. (F) Bacterial titers in the colon (n=4–5). (G) IFNγ expression in the colon was measured by real-time PCR (n=13–15). (H) Representative flow cytometry plots of IFNγ-producing ILCs (Lin^−^ Thy1^+^Eomes^−^) after stimulation with PMA and ionomycin; Flow plots show percentage of IFNγ^+^ cells among ILCs; (I) Frequency and total cell numbers of IFNγ^+^ ILCs (n=6–7). (K) Frequency and total cell numbers of monocytes (Ly6G^−^ MHCII^−^ CD64^+^CD11b^+^). (L) Frequency and total cell numbers of macrophages (Ly6G^−^ MHCII^+^CD64^+^CD11b^+^). Each symbol represents individual mouse. (A, I-L) Data are pooled from two independent experiments. (C, G) Data are pooled from three independent experiments. (D-F) Data is representative of one out of three independent experiments. Data shown as mean±SEM. ns- not significant, *p<0.05, **p<0.01, ***p<0.001. (C) one-way ANOVA with Dunnett’s multiple comparisons test, (A, D-L) two-tailed unpaired *t*-test.

As tofacitinib treatment inhibited IFNγ production by both T cells and ILCs, we next tested whether inhibition of JAK/STAT pathway is sufficient to ameliorate intestinal inflammation independently of its effects on T cells. To address this, we blocked IL-10R signaling in T-cell deficient TCRβδ^−/−^ mice, infected them with *C. jejuni*, and treated with tofacitinib as shown in Fig. 7B. Tofacitinib-treated TCRβδ^−/−^ mice displayed less body weight loss and reduced colon thickening compared to vehicle-treated mice (Fig. 8A and C). Consistently, histological analysis demonstrated ameliorated intestinal damage after tofacitinib treatment (Fig. 8D and E). Importantly, tofacitinib treatment did not affect *C. jejuni* colonization in the colon of TCRβδ^−/−^ mice (Fig. 8F). Moreover, attenuated intestinal pathology in tofacitinib-treated group was accompanied by reduced levels of IFNγ, IL-12 and IL-1β in the colon, whereas TNF, IL-6 and IL-22 levels were not affected by tofacitinib treatment (Fig. 8G and Fig. S7A).

Flow cytometry analysis revealed reduced IFNγ production by ILCs in the absence of T cells (Fig. 8H and I). The frequency of ILCs in tofacitinib-treated mice was comparable to vehicle-treated mice, whereas the numbers of ILCs in the colon were significantly reduced after tofacitinib treatment (Fig. S7B). Tofacitinib treatment significantly reduced the accumulation of monocytes and macrophages in the colon after *C. jejuni* infection (Fig. 8K and L). Although there was a trend towards the reduction of neutrophil numbers in tofacitinib-treated group, it was not significant (Fig. S7C). These data suggests that tofacitinib treatment ameliorates *C. jejuni*-induced intestinal pathology driven by innate immune cells via inhibiting of monocyte and macrophage accumulation in the colon and via reduction of IFNγ production by ILCs.

## Discussion

While tofacitinib-driven inhibition of the JAK/STAT pathway leads to reduced inflammation, it can also impair immune response against infections.^1,2^ However, the impact of tofacitinib on mucosal immunity against enteric pathogens remains poorly understood. Our findings demonstrate that tofacitinib treatment early during infection ameliorates intestinal inflammation driven by human mucosal pathogen *C. jejuni* in both IL-10 KO mice and in humanized mice with intact IL-10 signaling. This treatment limits the recruitment of circulating monocytes to the colon and suppresses IFNγ production by ILCs and T cells in response to infection. Importantly, we found that while JAK/STAT inhibition reduced inflammation, it had no effect on *C. jejuni* colonization in the colon. Although tofacitinib suppressed IFNγ production by NK1.1^−^ ILC1s and ex-ILC3s derived from NCR^−^ progenitors, it did not affect ILC3 to ILC1 plasticity. Furthermore, the effects of tofacitinib were restricted to the colon and did not affect immune response in mesenteric lymph node. These results provide insights into tofacitinib effects on mucosal immune response to intestinal bacterial infections.

Our data indicate that tofacitinib treatment early during infection ameliorated *C. jejuni*-induced intestinal inflammation, while treatment during established disease had no significant impact, suggesting that alternate inflammatory pathways may dominate during later stages of campylobacteriosis. Tofacitinib had no effect on bacterial colonization in the colon or on the expression of S100a8, an antibacterial protein known to contribute to protection against *C. jejuni*.^20,21^ This aligns with previous report indicating that tofacitinib had limited effect on S100a8 expression in primary human keratinocytes.^7^

Although tofacitinib reduced the expression of Muc1, a mucin implicated in limiting *C. jejuni* colonization and systemic dissemination,^20^ it did not change the bacterial load in the intestine. It is plausible that reduced Muc1 expression reflects dampened intestinal inflammation and reduced immune cell infiltration rather than impaired epithelial barrier function. Although our results suggest that tofacitinib treatment does not compromise anti-bacterial response to *C. jejuni*, the effects of tofacitinib to other intestinal pathogens remain to be determined.

The JAK/STAT pathway contributes to the maintenance of the intestinal epithelial barrier.^24,40^ *C. jejuni* invasion induces intestinal epithelial barrier disruption, leading to proinflammatory cytokine production.^24,41,42^ IL-6 can activate STAT3 in an autocrine manner via the JAK1/2 pathway.^16,43^ We found that *C. jejuni* infection upregulated IL-6 production in CMT-93 epithelial cells, whereas tofacitinib abolished IL-6 production and reduced STAT3 phosphorylation. IL-22 also activates STAT3 in epithelial cells to promote tissue repair.^24^ Previous studies have shown that tofacitinib inhibits IL-22-driven STAT3 phosphorylation in human colonic epithelial organoids and impairs epithelial repair in a DSS-colitis model.^44,45^ However, in *C. jejuni* colitis model, IL-22 expression remained unchanged in the colon tissue in both tofacitinib- and vehicle-treated groups. Correspondingly, tofacitinib did not alter STAT3 phosphorylation in IEC, although it slightly reduced pSTAT3 in cLP cells early at day 3 pi. These findings suggest that tofacitinib primarily inhibits STAT3 activation in immune cells in the cLP early after infection.

*C. jejuni* intestinal pathology is driven by IL-17 and IFNγ signaling, while IL-22 is dispensable.^13,14,19^ Consistently, IL-22 deficiency did not exacerbate intestinal pathology following *C. jejuni* infection.^19^ Tofacitinib treatment reduced IFNγ levels in the colon, along with decreased JAK-dependent STAT1 phosphorylation, whereas IL-17 expression remained unaffected. These results indicate that tofacitinib alleviates *C. jejuni*-induced intestinal pathology primarily via inhibition of IFNγ without affecting IL-17 and IL-22 signaling.

We also found that early tofacitinib treatment mitigated colonic inflammation in mice infected with *C. difficile*. As IL-22 is critical for epithelial cell regeneration in *C. difficile* colitis,^46^ inhibiting JAK/STAT signaling during later stages of infection may impair mucosal healing, as previously reported in DSS-induced colitis.^45^ Therefore, further studies are needed to define how JAK/STAT inhibition affects epithelial repair during *C. difficile* and other enteric infections. This will help further define therapeutic windows that suppress inflammation without impairing mucosal healing.

Neutrophils and mononuclear phagocytes are the first immune cells to respond to intestinal infection by eliminating pathogen and orchestrating downstream inflammation. Consistent with previous reports,^13,47^ we found an influx of neutrophils, monocytes, and macrophages in the colon of *C. jejuni* infected mice. Notably, tofacitinib treatment significantly reduced neutrophil accumulation in the colon, which correlated with decreased expression of the chemokines CXCL1 and CXCL2. Tofacitinib also impaired monocyte recruitment to the colon, at least in part, by reducing circulating monocytes during the early stage of *C. jejuni* infection. Whether JAK/STAT inhibition impairs monocyte development in the bone marrow remains to be determined.

A prior study indicated that tofacitinib regulates interferon-responsive gene expression in macrophages in secondary lymphoid organs,^48^ although the specific roles of monocytes and macrophages in *C. jejuni*-induced intestinal pathology has not been studied. Our *in vitro* experiments using human THP-1 cell line demonstrated that JAK/STAT inhibition dampens *C. jejuni*-induced production of IL-6 and IL-12. Based on these findings, we propose that tofacitinib may limit monocyte migration from the bloodstream to the colon and hinder their differentiation into macrophages, thereby reducing the production of IFNγ-promoting IL-6 and IL-12 cytokines (Fig. S8). Additionally, our *in vitro* data suggest that tofacitinib decreased CCL2 production by both epithelial cells and monocytes, which may contribute to the reduced monocyte infiltration during *C. jejuni* infection.

Our results suggest that tofacitinib effects are tissue-dependent, as it reduced intestinal inflammation and IFNγ production in the colon but not in MLN in both *C. jejuni* infected IL-10 KO mice and in humanized mice. This could be due to rapid tofacitinib absorption and distribution in the gastrointestinal tract^49,50^ with limited systemic effects. In our study we treated mice twice daily with the 30 mg/kg dose that mimics clinical dose used in human studies.^48^ In humans, the predicted gut availability of tofacitinib was more than 90%, suggesting that the intestine is the main site of action when the inhibitor is orally administered.^50^ A recent study showed that systemic and tissue concentrations of the JAK/STAT inhibitors can be affected by the severity of intestinal inflammation,^51^ yet more studies are needed to fully understand the influence of inflammatory immune responses on pharmacokinetics and disposition of JAK/STAT inhibitors in the mucosal tissues.

*C. jejuni*–mediated intestinal pathology during the early stage of infection is driven by innate immune response.^47,52^ IFNγ-producing ILCs contribute to *C. jejuni*-driven colitis.^14^ Our results demonstrate that tofacitinib treatment ameliorates intestinal pathology, similarly to studies using IFNγ depletion.^13,14^ Since tofacitinib ameliorated intestinal pathology and reduced IFNγ expression in the colon, we focused on IFNγ-producing cells. A previous study showed that tofacitinib impairs IFNγ production by human ILCs stimulated with IL-12 and IL-15 *in vitro*.^17^ However, how JAK/STAT inhibitors block effector functions of ILCs in bacteria-induced colitis remained unclear. Our data corroborate these findings and demonstrate that tofacitinib inhibited intestinal IFNγ production by ILCs during *C. jejuni*-induced colitis. ILCs which express natural killer surface markers NK1.1 and NKp46 (NCR^+^) secrete high levels of IFNγ upon activation.^33,34^ Previously we showed that *C. jejuni* infection drives expansion of ILCs that do not express NK1.1.^14^ Although both NCR^+^ and NCR^−^ ILCs produce IFNγ upon *C. jejuni* infection,^14^ our data suggest that NCR^−^ ILCs produce more IFNγ compared to NCR^+^ ILCs. This is consistent with a previous study suggesting that NCR^−^ ILCs have a potency to produce more IFNγ than NCR^+^ ILCs in the gut during *Yersinia enterocolitica* infection.^53^ NCR expression in intestinal ILCs is not stable, as some ILCs downregulate NCRs during differentiation, to become double-negative for NK1.1 and NCR.^33^ Our fate-mapping analysis suggests that while the majority of IFNγ-producing ILCs originate from NKp46^+^ cells in the colon of uninfected mice, *C. jejuni* infection induces IFNγ production predominantly by ILCs that derive from distinct NKp46^−^ progenitor cells. In line with our results, other reports indicate that NKp46 expression is not necessary for IFNγ production by ILC1s in other infection models, as IFNγ production by ILC1s was preserved despite the genetic ablation of NKp46.^54,55^

Inflammatory conditions can induce ILC plasticity in the intestine.^34,38^ Although the JAK/STAT pathway is essential for ILC development and homeostasis, it remains unclear whether JAK/STAT inhibitors impair ILC functions during bacterial intestinal infections. We previously demonstrated that *C. jejuni* infection promotes T-bet^+^ ILC1s with a history of RORγt expression, suggesting the conversion of ILC3s to ILC1s (ex-ILC3s).^14^ In this study, we found that tofacitinib treatment reduced the number of NK1.1^−^ RORγt^fm+^ ILCs in the intestine which includes both RORγt^+^ ILC3s and ex-ILC3s populations. Moreover, tofacitinib impaired proliferation of NK1.1^−^ ILCs, which could explain more pronounced effect on these cells. As tofacitinib treatment primarily reduced the proliferation of NK1.1^−^ ILCs without affecting total ILC numbers in the gut, observed effect on RORγt^fm+^ ILCs during *C. jejuni* infection could be a consequence of altered proliferation rather than changes in plasticity.

IL-10 drives anti-inflammatory responses by suppressing excessive inflammation in a STAT3-dependent manner.^56^ We showed that tofacitinib reduced STAT3 phosphorylation in the colon of IL-10 KO mice after *C. jejuni* infection. The impact of JAK/STAT suppression on IL-10 signaling is controversial.^57-61^ In one study, tofacitinib treatment decreased IFNγ and IL-6 but increased IL-10 production by monocytes from IBD patients.^57^ Conversely, other studies demonstrated increased production of proinflammatory cytokines along with IL-10 by human monocytes and mouse macrophages following tofacitinib treatment.^58,61^ These findings suggest that JAK/STAT pathway effects on pro- and anti-inflammatory responses vary by cell type and condition. Therefore, we wanted to know whether our data obtained in IL-10 KO mice treated with tofacitinib could be translated to mice with human immune system and functional IL-10 signaling. Our results demonstrate that *C. jejuni* infection induced human IFNγ production in the colon of THX and hu-PBMC-NSG humanized mice, consistent with increased IFNγ levels in *C. jejuni*-infected human patients.^62^ Although the intestinal inflammation driven by *C. jejuni* was mild in humanized mice, it recapitulates the self-limiting infection during human campylobacteriosis in healthy individuals. In THX mice, tofacitinib reduced IFNγ production in the colon without affecting host protection, suggesting that its effects on IFNγ production during *C. jejuni* infection are independent of IL-10 signaling in our experimental settings.

Collectively, our study highlights the critical role of the JAK/STAT pathway in mediating IFNγ-driven intestinal pathology during *C. jejuni* infection. We propose that tofacitinib alleviates *C. jejuni*-induced colitis through multiple mechanisms (Fig. S8). Specifically, tofacitinib suppresses JAK/STAT-dependent IL-6 and IL-12 production by monocytes and macrophages, leading to reduced IFNγ production by ILC1s and T cells. Additionally, it inhibits neutrophil recruitment to the intestine by inhibiting CXCL1 and CXCL2 chemokine production from intestinal epithelial cells. Tofacitinib may also directly suppress IFNγ production by ILCs and T cells.

Recent studies have shown that a *C. jejuni* genotoxin can activate the JAK2/STAT3 axis in colorectal tumors, exacerbating lung and liver metastasis.^63^ Our findings further suggest that JAK/STAT signaling contributes to *C. jejuni*-induced colitis. Given that colitis can drive colorectal cancer progression, these results raise the possibility that JAK/STAT signaling may play a role in the development of *C. jejuni*-associated colorectal cancer. Further research is warranted to elucidate how *C. jejuni* activates JAK/STAT signaling in both inflammatory and neoplastic contexts, which may inform future therapeutic strategies aimed at preventing metastasis in *C. jejuni*-positive colorectal cancer.

Importantly, our results also demonstrate that tofacitinib suppresses the effector functions of ILCs in response to *C. jejuni* infection without compromising antibacterial immunity. Since ILCs are the key regulators of intestinal inflammation in IBD, understanding how JAK/STAT inhibitors modulate ILC function could provide valuable insights for optimizing IBD treatment strategies.

## Methods

### Mice

All animal studies were conducted in accordance with the University of Texas Health Science Center at San Antonio Animal Care and Use guidelines and approved by Institutional Animal Care and Use Committee (Protocol #20170020AR). Ten to twelve-week-old male and female mice were used for experiments. C57BL/6 mice were originally obtained from The Jackson Laboratory (Bar Harbor) and bred at the University of Texas Health Science Center at San Antonio. IL-10 deficient mice (B6.129P2-*Il10*^*tm1Cgn*^/J),^64^ IFNγ reporter mice (B6.129S4-*Ifng*^*tm3.1Lky*^/J)^65^ and TCRβδ^−/−^ (TCR B6.129P2-*Tcrb*^*tm1Mom*^*Tcrd*^*tm1Mom*^/J)^66^ were reported previously. To generate RORγt (RORγt^fm^) or NKp46 (NKp46^fm^) cell fate-mapping mice, mice expressing EYFP or TdTomato that is preceded by a loxP-flanked STOP sequence in the Rosa26 locus^36^ were intercrossed with RORγt-Cre^39^ or NKp46-Cre mice.^35^ All mice used in this study were on C57BL/6 background and maintained under specific pathogen free conditions. Age/sex matched controls or littermate controls were used for all experiments. Our study examined males and females, and similar findings are reported for both sexes.

### Human immune system mice models

NOD.Cg-*Prkdc*^*scid*^*Il2rg*^*tm1Wjl*^/SzJ (NSG) mice engrafted with human peripheral blood mononuclear cells (PBMC)(#74557) were obtained from The Jackson Laboratory (Bar Harbor). THX humanized (NOD.Cg-*Kit*^*W*–41*J*^ *Tyr* ^+^ *Prkdc*^*scid*^ *Il2rg*^*tm1Wjl*^/ThomJ) mice engrafted with human cord blood CD34^+^ cells were recently developed.^32^

### C. jejuni infection mouse model

10–12 weeks old mice were pretreated with antibiotic cocktail for 7 days in drinking water as previously described.^47^ Antibiotics were changed to water one day prior to infection. Mice were inoculated by oral gavage with a single dose of either Mueller Hinton Broth (mock) or 1–5 × 10^9^ colony-forming unit (CFU) of *C. jejuni* (NCTC 11168) in 0.4 ml volume as described previously.^14^ The infection dose was confirmed for each experiment by plating serial dilutions of bacterial stock onto Mueller Hinton agar (M–H agar). To enumerate bacterial loads, serial dilutions of colon homogenates were plated on M–H agar as previously described,^67^ colonies were counted after 48 h of incubation at 42°C under microaerophilic conditions using AnaeroJars and Oxoid Campy-Gen sachets (Thermo Scientific).

### C. difficile infection

*Clostridioides difficile* R20291 spores were prepared, as described.^22^ 8–10 week old age-matched and sex-matched C57BL/6 mice were pretreated with antibiotic cocktail in drinking water for 4 days as previously described.^68^ 24 h after single dose clindamycin (10 mg/kg i.p. Sigma), mice were orally infected with 1 × 10^8^ CFU of *C. difficile* spores.

### In vivo anti-IL-10Ra treatment

To block IL-10R signaling *in vivo*, mice were treated i.p. with 200 μg of aIL-10Ra (clone 1B1.3A, BioXcell) prior to infection, one day after infection and then every 3 days.

### Jak inhibitor

Tofacitinib (CP-690550 from Pfizer or LC lab) was resuspended in 0.5% methylcellulose/0.025% Tween 20 (Sigma) for *in vivo* studies or in DMSO for *in vitro* use. To inhibit the JAK/STAT pathway *in vivo*, mice were orally treated twice daily with vehicle (0.5% methylcellulose/0.025% Tween 20) or 30 mg/kg of tofacitinib starting next day after *C. jejuni* inoculation.

### Histology

Dissected colons were fixed in 10% neutral buffered formalin (Fisher Scientific), paraffin-embedded, sectioned, and stained with H&E for histological evaluation. Images were taken with Zeiss Axiophot2 or Keyence BZ-X800 microscope. Intestinal inflammation was graded as previously described.^19^

### Cell isolation

Colonic cells from intraepithelial and lamina propria fractions were isolated as previously described.^69^ Briefly, colon and cecum were harvested, and intestines were cut longitudinally, washed with cold PBS. To remove epithelial cells, tissues were cut in̴ 1 cm pieces and incubated in RPMI 1640 media containing 3% FBS and 5 mM EDTA at 37°C for 20 min with 150 rpm shaking. After incubation, cells were vortexed in RPMI 1640 media containing 2 mM EDTA, supernatants containing IELs, were kept for further isolation. Colon tissue pieces were transferred to digestion buffer containing 200 μg/ml Liberase (Roche), 0.05% DNAse I (Sigma) and incubated at 37°C for 40 min with 150 rpm shaking. Leukocytes from IEL and LP were enriched by 40/80% Percoll (Cytiva) gradient centrifugation. Single cell suspension of mLN were prepared by passing through 70 μm cell strainer and used for staining.

Epithelial cells were isolated as previously described.^70^ For epithelial cell isolation, small pieces of colon and cecum tissues were incubated in ice-cold PBS with 30 mM EDTA with vigorous shaking every 8–10 min. After a single wash in cold PBS, tissues were digested in TrypLE Express (Gibco) for 10 min at RT. The single cell suspension was passed through a 70 μm strainer. Cells were used for staining or western blot analysis.

### Flow cytometry

Cell staining was performed using standard protocol (details can be found in the Supplemental Experimental Procedures). Flow cytometry data were collected using an LSRII (BD Biosciences) or Cytek Aurora (Cytek Biosciences), and analyzed using FlowJo 10 software (FlowJo LLC).

### Real-time RT-PCR analysis

RNA from colon and mLN was isolated using E.Z.N.A. Total RNA kit I (Omega Bio-tek). cDNA synthesis and real-time RT-PCR was performed as described previously^71^ using Power SYBR Green master mix (Applied Biosystems). Relative mRNA expression of the target genes was determined using the comparative 2^−ΔΔCt^ method. Primers used are listed in Supplementary Experimental Procedures Table 1 and 2. Data normalized to *hprt* expression unless stated otherwise.

### Western blotting

STATs analysis in the colon tissue or in THP-1/CMT-93 cells was performed using standard protocol (details can be found in the Supplemental Experimental Procedures).

### In vitro cultures

Colon lamina propria cells from IFNγ-reporter mice were isolated after 6 days of *C. jejuni* infection. Cells were stimulated in RPMI 1640 containing 10% FBS, 50 ng/ml PMA and 750 ng/ml ionomycin in the presence of tofacitinib (200 nM or 1000 nM) or vehicle, at 37°C in 5% CO_2_. After 16 h of incubation, 10 μg/ml of Brefeldin A was added to the cells. The cells were surface stained and analyzed by flow cytometry after 18 h of incubation with tofacitinib or vehicle.

### Cell culture

Mouse rectal epithelial cells CMT-93 (ATCC CCL-223) were cultured in DMEM (Corning) media supplemented with heat-inactivated 10% fetal bovine serum (FBS).

Human monocytic cell line THP-1 (ATCC TIB-202) were grown in RPMI 1640 (Corning) supplemented with heat-inactivated 10% FBS, 10 mM HEPES and 0.05 mM 2-mercaptoethanol. Cells were cultured at 37°C in 5% CO_2_. (details can be found in the Supplemental Experimental Procedures).

### Statistical analysis

Statistical analysis was performed using two-tailed *t* test, one-way or two-way analysis of variance (ANOVA) when appropriate. Data are presented as means±S.E.M. with p*<*0.05 being considered significant. All statistical tests were performed in GraphPad Prism 10.0 program. ns – not significant, *: p<0.05, **: p<0.01, ***: p<0.001.

## Supplementary Material

Supplementary Material

Supplementary data to this article can be found online at 10.1016/j.mucimm.2025.06.010.

## Data Availability

The authors confirm that the data supporting the findings of this study are available within the article and its Supplementary materials. There are no large datasets requiring deposition in a public repository.
