# Supplementary material for: Tofacitinib ameliorates Campylobacter-induced intestinal pathology by suppressing IFNγ producing ILCs and T cells
Source: Mucosal Immunol. Author manuscript; Available in PMC 2025 Sep 13. (PMC12431686; doi:10.1016/j.mucimm.2025.06.010)
Supplement: Supplementary Material [file NIHMS2107220-supplement-Supplementary_Material.pdf]

Figure S1. Tofacitinib limits early *C. jejuni- and C. difficile*-induced intestinal pathology. (A) IL-10 KO mice were infected with *C. jejuni* and treated with tofacitinib (Tofa), starting the day after infection for 5 days (d1-5). **(B)** IL-10 KO mice were infected with *C. jejuni* and treated with Tofa at days 5 to 8 pi. On day 9 pi **(C)** colon mass-to-length and **(D)** bacterial loads were assessed (n=4-7). **(E-H)** C57BL/6 mice were infected with 10^8 CFU of *C. difficile* spores by oral gavage after antibiotics treatment according to the schematic. **(F)** Mice were monitored for body weight loss for 5 days. **(H)** Colon length (n=3-4). Each symbol represents individual mouse. **(B-D)** Data are pooled from two independent experiments. **(F-H)** Data is representative of one out of two independent experiments with similar results. Data shown as mean ± SEM. ns- not significant, *p<0.05. **(A-B, F, H)** two-tailed unpaired t-test or **(C-D)** one-way ANOVA with Tukey's multiple comparisons test.

Figure S2. Effect of tofacitinib treatment on intestinal epithelial cells during *C. jejuni* infection. (A) Schematic of the experiment. Cells from blood, colon lamina propria (cLP) and intestinal epithelial cells (IEC) were isolated from *C. jejuni* infected IL-10 KO mice on day 3 pi **(B)** Colon length (n=5-6); **(C)** Bacterial loads in the colon (n=5-6); **(D)** Representative flow cytometry plot and frequency of IEC ($CD45^-$ $CD31^-$ $Ter119^-$ $EpCAM^+$); **(E)** Frequency of $Ki\text{-}67^+$ IEC (n=5-7); **(F)** Expression of Ki-67 in the colon (n=5-6). Data were normalized to *hprt* expression. **(G-H)** Representative blots of pSTAT3/STAT3 and relative density of pSTAT3 to STAT3 in (**G**) IEC and **(H)** cLP. **(I-L)** CMT-93 cells were mock-treated or *C. jejuni*-infected for 4 h, then given tofacitinib (Tofa) or vehicle and incubated for 16 h before analysis. **(J-K)** Expression of proinflammatory cytokines (**J**) and chemokines **(K)** (n=3-5). **(L)** Representative blots of pSTAT3 and STAT3 following *C. jejuni* infection and treatment with Tofa (n=2-3/group). **(B-H)** Each symbol represents an individual mouse. **(J-K)** Each symbol represents a biological replicate. **(B-F, J-L)** Data are pooled from two independent experiments. **(G-H, L)** Data is representative of one out of two independent experiments. Data shown as mean ± SEM. ns- not significant, *p<0.05, **p<0.01, ***p<0.001. **(A-C, D-H)** two-tailed unpaired t-test or **(J-K)** one-way ANOVA with Dunnett's multiple comparisons test.

Figure S3. Analysis of myeloid cell population in the colon of *C. jejuni*-infected IL-10 KO mice after tofacitinib treatment. IL-10 KO mice were treated according to the schematic on Fig 1a. Cells from colon lamina propria were isolated from mock or *C. jejuni* infected IL-10 KO mice on day 6 p.i. Gating strategy to identify neutrophils ($CD45^+$ $Ly6G^+$), monocytes ($CD45^+$ $Ly6G^-$ $MHCII^-$ $CD11b^+$ $CD64^+$), macrophages ($CD45^+$ $Ly6G^-$ $MHCII^+$ $CD11b^+$ $CD64^+$) and dendritic cells ($CD45^+$ $Ly6G^-$ $MHCII^+$ $CD64^-$ $CD11c^+$).

Figure S4. JAK/STAT inhibition reduces monocytes in *C. jejuni* infected IL-10 KO mice. (A) Schematic of the experiment. Cells from blood, colon lamina propria (cLP) were isolated from *C. jejuni* infected IL-10 KO mice on day 3 pi **(B)** Frequency of neutrophils ($Ly6G^+$ $CD11b^+$) and monocytes ($MHCII^-CD11b^+$ $CCR2^+$ $Ly6C^+$) in the blood. **(C)** Frequency and cell numbers of neutrophils ($Ly6G^+$ $CD11b^+$), monocytes ($Ly6G^-$ $MHCII^-$ $CD11b^+$ $CD64^+$) and macrophages ($Ly6G^-$ $MHCII^+$ $CD11b^+$ $CD64^+$) in cLP. Each symbol represents an individual mouse. **(B-C)** Data are pooled from two independent experiments. Data shown as mean ± SEM. ns- not significant, *p<0.05, **p<0.01, **(B)** one-way ANOVA with Dunnett's multiple comparisons test or **(C)** two-tailed unpaired t-test.

Figure S5. Analysis of ILCs and T cells in the colon of *C. jejuni*-infected IL-10 KO mice after tofacitinib treatment. IL-10 KO mice were treated according to the schematic on Fig 1a. Cells from colon lamina propria were isolated from mock or *C. jejuni* infected IL-10 KO mice on day 6 pi. For IFNγ staining, cells were stimulated with PMA and ionomycin in the presence of Brefeldin A for 4h. **(A)** Gating strategy to identify T cells ($CD3^+$), ILCs ($CD3^-Lin^-Thy1.2^+Eomes^-$) and NK cells ($CD3^-Eomes^+T\text{-}bet^+$ $NK1.1^+$) in the colon and mLN; **(B)** Total cell numbers of $IFN\gamma^+$ NK cells (n=6-7). **(C)** Frequency and total cell numbers of T cells (n=6-7). **(D)** Total cell numbers and frequency of ILCs (n=6-7). Frequency of ILCs shown among CD3- cells. **(E)** Total cell numbers of NK cells (n=6-7). **(F)** Total cell numbers of T cells (left panel) and ILCs (right panel) in the mLN. **(G)** Representative flow cytometry plots of IFNγ- producing ILC3s ($CD3^-Lin^-CD90.2^+Eomes^-ROR\gamma t^+$). Flow plots show the percentage of $IFN\gamma^+$ cells among ILCs. **(H)** Frequency and total cell numbers of $IFN\gamma^+$ ILC3 (n=3-6); Lineage- (Lin^-): $B220^-CD11c^-Ter119^-Gr1^-CD5^-$. **(I)** Representative plot of IFNγ-production by ILC2s ($KLRG1^+$ $T\text{-}bet^+$) and ILC1s ($T\text{-}bet^+$ $KLRG1^-$) in the colon; Each symbol represents individual mouse. Data are pooled from two independent experiments **(B-E; H)**. Data is representative of one out of three independent experiments **(F)**. Data shown as mean ± SEM. ns- not significant, *p<0.05, **p<0.01. (**B-F, H**) one-way ANOVA with Dunnett's multiple comparisons test.

Figure S6. Tofacitinib impairs IFNγ production by ILCs derived from NCR$^-$ progenitors during *C. jejuni* infection. (A) IL-10 KO mice were treated according to the schematic on Fig 1A. Colon lamina propria cells were isolated from *C. jejuni* infected vehicle-treated or Tofa-treated mice on day 6 pi. Frequency and total cell numbers of IFNγ-producing NKp46$^+$ and NKp46$^-$ ILCs (n=3-4). **(B-E)** The history of NKp46 expression was determined in NKp46^{fm} mice. **(B)** Schematic of experimental design. NKp46^{fm} mice were treated with 200 µg of IL-10Rα blocking mAb (clone 1B1.3A from BioXCell, i.p.) on days -1, 1, 4 and 7 after mock or *C. jejuni* (*C.j*) infection. Cells from the colon lamina propria were isolated on day 10 pi. Cells were stimulated with PMA and ionomycin in the presence of Brefeldin A. **(C)** Representative flow cytometry plots of IFNγ-producing NKp46^{fm+} (green) and NKp46^{fm-} (yellow) ILCs (CD3$^-$Lin$^-$Thy1.2$^+$). Flow plots show percentage of IFNγ-producing NKp46^{fm+} and NKp46$^-$ cells among ILCs. **(D)** Frequency and total cell numbers of NKp46^{fm-} ILCs (n=2); **(E)** Frequency and total cell numbers of NKp46^{fm+} ILCs (n=2); Lineage: B220, CD11c, Ter119, Gr1, CD5. Each symbol represents an individual mouse. Data is representative of one out of three independent experiments. Data shown as mean ± SEM. ns- not significant, *p<0.05, **p<0.01. **(A)** two-way ANOVA with Tukey's multiple comparisons test, **(D-E)** two-tailed unpaired t-test.

Figure S7. Cytokine expression and immune cell populations in the colon of *C. jejuni*-infected T-cell deficient mice after tofacitinib treatment. TCRβδ$^{-/-}$ mice were treated according to the schematic on Fig 7b. **(A)** Expression of cytokines was measured by real-time PCR (n=13-15). Data were normalized to *hprt* expression. **(B)** Total cell numbers and frequency of ILCs (Lin$^-$Thy1.2$^+$) (n=6-7)); **(C)** Total cell numbers and frequency of neutrophils (CD45$^+$Ly6G$^+$) (n=6-7). Data are pooled from two independent experiments. Data shown as mean ± SEM. ns- not significant, *p<0.05, **p<0.01, ***p<0.001, two-tailed unpaired t-test.

Figure S8. Model. Tofacitinib ameliorates *C. jejuni*-induced intestinal pathology without compromising host protection. *C. jejuni* infection of monocyte/macrophages leads to STAT3 activation and production of IL-12/IL-6 which promote IFNγ production by ILC1s and T cells thereby leading to intestinal pathology. Tofacitinib ameliorates *C. jejuni*-induced colitis by multiple mechanisms: 1) inhibiting production of the neutrophil-recruiting chemokines CXCL1/2 by intestinal epithelial cells; 2) reducing IL-12/IL-6 secretion by monocytes/macrophages thereby suppressing IFNγ production by T cells and ILC1s. 3) directly suppressing IFNγ production by T cells and ILC1s. Tofacitinib does not compromise bacterial colonization. Figure was created with BioRender.com.

Supplemental Experimental Procedures

Flow cytometry. Before surface staining single cell suspension were incubated with anti-CD16/32 antibody (BioXCell, 2.4G2) for 20 min on ice. Dead cells were excluded using Zombie NIR™ Fixable Viability (Biolegend) staining. Thereafter, cells were stained for surface antigens for 30 min on ice. Flow cytometry antibodies used for surface staining to mice or human proteins were purchased from Biolegend and eBioscience : Anti-CD45 (104), anti-EpCAM (68.8), anti-CD3 (145-2C11), anti-Thy1.2 (30-H12), anti-B220 (RA3-6B2), anti-Ter-119 (TER119), anti-Gr-1 (RB6-8C5), anti-CD11c (N418), anti-CD5 (53-7.3), anti-Ly6G (1A8), anti-CD11b (M1/70), anti-CD64 (X54-5/7.1), anti-CD31 (MEC13.3), anti-MHCII (M5/114.15.2), anti-Ly6C (HK1.4), anti-KLRG1 (2F1), anti-NK1.1 (PK136), anti-NKp46 (29A1.4), anti-CCR2 (475301, BD OptiBuild). Lineage- for ILCs staining: CD5, CD11c. B220, Ter119, Gr-1. For transcription factors staining, cells were first stained for surface markers, fixed and permeabilized using True-NuclearTM transcription factor buffer set (Biolegend) according to the manufacturer's protocol. For transcription factors staining following antibodies were used: anti-Eomes (Dan11mag, eBioscience), anti-T-bet (4B10, Biolegend), anti-RORγt (Q31-378, BD Pharmigen), anti-Ki-67 (16A8). For IFNγ evaluation prior to staining cells were stimulated in PRMI 1640 containing 10% FBS, 50 ng/ml PMA and 750 ng/ml ionomycin in the presence of 10 microgram/ml of Brefeldin A for 4 h. After surface staining cells were fixed and permeabilized. For intracellular mouse cytokines, anti-IFNγ (XMG1.2) antibodies from Biolegend were used.

For staining of human proteins in humanized mice, prior to surface staining cells were incubated with human TruStain FcX™ (Biolegend) for 15 min at RT. Dead cells were excluded by eBioscience™ Fixable Viability Dye eFluor™ 450. For surface staining antibodies from Biolegend were used: anti-hCD45 (H130), anti-hCD3 (OKT3), anti-hCD4 (OKT4). For human IFNγ staining cells were stimulated with PMA/Ionomycin and Brefeldin as described earlier and stained with anti-hIFNγ (4S.B3) from Biolegend. Flow cytometry data were collected using an LSRII (BD Biosciences) or Cytek Aurora (Cytek Biosciences), and analyzed using FlowJo 10 software (FlowJo LLC).

Western blotting. Whole colon tissues were lysed in RIPA buffer (Thermo Scientific) containing cocktail of proteases (Thermo Scientific) and phosphatases inhibitors (Sigma). The protein concentration was measured using Pierce BCA protein assay kit. Equal amounts of total protein were separated by SDS-PAGE, transferred to PVDF membrane (0.2 µm, Invitrogen) and blocked with 5% BSA in TBST buffer (Tris-buffered saline, 0.1% Tween 20) at 4°C overnight. Membranes were washed and incubated with primary antibodies (Cell Signaling technology or Abcam): STAT3 (79D7), pSTAT3-Tyr705 (D3A7), STAT1 (D4Y6Z), pSTAT1 (58D6) and anti-GAPDH (mAbcam 9484). The goat anti-rabbit IgG-HPR (Kindle Biosciences) and anti-mouse IgG-HPR (Southern Biotechnology) were used for detection by chemiluminescent detection with Pierce ECL Western Blotting Substrate (Thermo Scientific). The bands were visualized by ChemiDoc MP Imaging system (Bio-Rad). The signal intensities of bands were quantified using ImageJ software and normalized relative to the intensity of loading control GAPDH.

Inoculation protocol. Bacterial preparation. *C. jejuni* (NCTC 11168) for cells inoculation was prepared as previously described [1,2] with minor modifications. *C. jejuni* was harvested from the M-H agar plates in sterile PBS and pelleted by centrifugation at 5000g for 10 min at 4°C. The OD at 600nm was measured. The pellets were washed in PBS, and bacteria were resuspended in antibiotic-free DMEM or RPMI 1640 media supplemented with 1% of FBS to achieve multiplicity of infection (MOI) of 100-250. MOI was confirmed by plating serial dilutions on M-H agar plates and colonies were counted after 48h incubation at 42°C under microaerophilic conditions using AnaeroJars and Oxoid CampyGen sachets (Thermo Scientific).

Inoculation and tofacitinib treatment. THP-1 monocytes or CMT-93 epithelial cells were seeded into 24- or 6 -well plates. CMT-93 cells were grown to monolayers. The day before bacteria inoculation, the culture medium was changed to antibiotic-free DMEM (CMT-93) or RPMI 1640 (THP-1) containing 1% FBS. THP-1 cells were infected with *C. jejuni* at MOI of 250, and CMT-93 cells at MOI of 100. After 4 h of infection, tofacitinib (100nM-1000nM) or vehicle was added. After incubation for 16h, cells were lysed in RNA lysis buffer for cytokines analysis or in RIPA buffer for western blot analysis.

References:

1. Jones, M.A., Tötemeyer, S., Maskell, D.J., Bryant, C.E., and Barrow, P.A. (2003). Induction of proinflammatory responses in the human monocytic cell line THP-1 by Campylobacter jejuni. Infection and immunity *71*, 2626–2633. 10.1128/iai.71.5.2626-2633.2003.

2. Siegesmund, A.M., Konkel, M.E., Klena, J.D., and Mixter, P.F. (2004). Campylobacter jejuni infection of differentiated THP-1 macrophages results in interleukin 1 beta release and caspase-1-independent apoptosis. Microbiology (Reading) *150*, 561–569. 10.1099/mic.0.26466-0.

Supplementary Table 1. Primers for quantitative PCR of mouse genes

Ifng FW	TCAAGTGGCATAGATGTGGAAGAA
Ifng RV	TGGCTCTGCAGGATTTTCATG
Il17a FW	CAGACTACCTCAACCGTTCCAC
Il17a RV	TCCAGCTTTCCCTCCGCATTGA
Tnf FW	ACGGCATGGATCTCAAAGAC
Tnf RV	AGATAGCAAATCGGCTGACG
Il22 FW	TCCGAGGAGTCAGTGCTAAA
Il22 RV	AGAACGTCTTCCAGGGTGAA
Il12b FW	ACAGCACCAGCTTCTTCATCA
Il12b RV	TCTTCAAAGGCTTCATCTGCAA
Il12a FW	ACGAGAGTTGCCTGGCTACTAG
Il12a RV	CCTCATAGATGCTACCAAGGCAC
Il6 FW	ACAAGTCGGAGGCTTAATTACACAT
Il6 RV	AATCAGAATTGCCATTGCACAA
Il1B FW	AAGCCTCGTGCTGTCGGACC
Il1B RV	TGAGGCCCAAGGCCACAGGT
Il23a FW	CAGCAGCTCTCTCGGAAT
Il23a RV	ACAACCATCTTCACACTGGATACG
Hprt FW	GCGTCGTGATTAGCGATGATGAAC
Hprt RV	CCAGTTTCACTAATGACACAAACG
Muc1 FW	ATGGGCAGCTGGACATCTTT
Muc1 RV	TGCCGAAACCTCCTCATAGG
S100a8 FW	TCCTCAGTTTGTGCAGAATATAAA
S100a8 RV	TCTTTGTGAGATGCCACACC
S100a9 FW	AAGGAAGGACACCCTGACAC
S100a9 RV	TCTTTCTTCATAAAGGTTGCCA
Cxcl1 FW	GCTGGGATTCACCTCAAGAA
Cxcl1 RV	TGGGGACACCTTTTAGCATC
Cxcl2 FW	CCTGGTTCAGAAAATCATCCA
Cxcl2 RV	CTTCCGTTGAGGGACAGC
Ccl2 FW	AACTCTCACTGAAGCCAGCTCT

Ccl2 RV	CGTTAACTGCATCTGGCTGA
TGFB1 FW	ACCATGCCAACTTCTGTCTG
TGFB1 RV	CGGGTTGTGTTGGTTGTAGA
Mki67 FW	GCCGAGTCTGGCATTGAA
Mki67 RV	TTTTCTTTCTTCTTTTGCTGAGG

Supplementary Table 2. Primers for quantitative PCR of human genes

Human *Hprt* FW	GACCAGTCAACAGGGGACAT
Human *Hprt* RV	GTGTCAATTATATCTTCCACAATCAAG
Human *Ifng* FW	TGGCTTTTCAGCTCTGCATC
Human *Ifng* RV	CCGCTACATCTGAATGACCTG
Human *Ccl2* FW	CATTGTGGCCAAGGAGATCTG
Human *Ccl2* RV	CTTCGGAGTTTGGGTTTGCTT
Human *IL6* FW	AGACAGCCACTCACCTCTTCAG
Human *IL6* RV	TTCTGCCAGTGCCTCTTTGCTG
Human *IL12A* FW	TGCCTTCACCACTCCCAAAACC
Human *IL12A* RV	CAATCTCTTCAGAAGTGCAAGGG

Fig S1

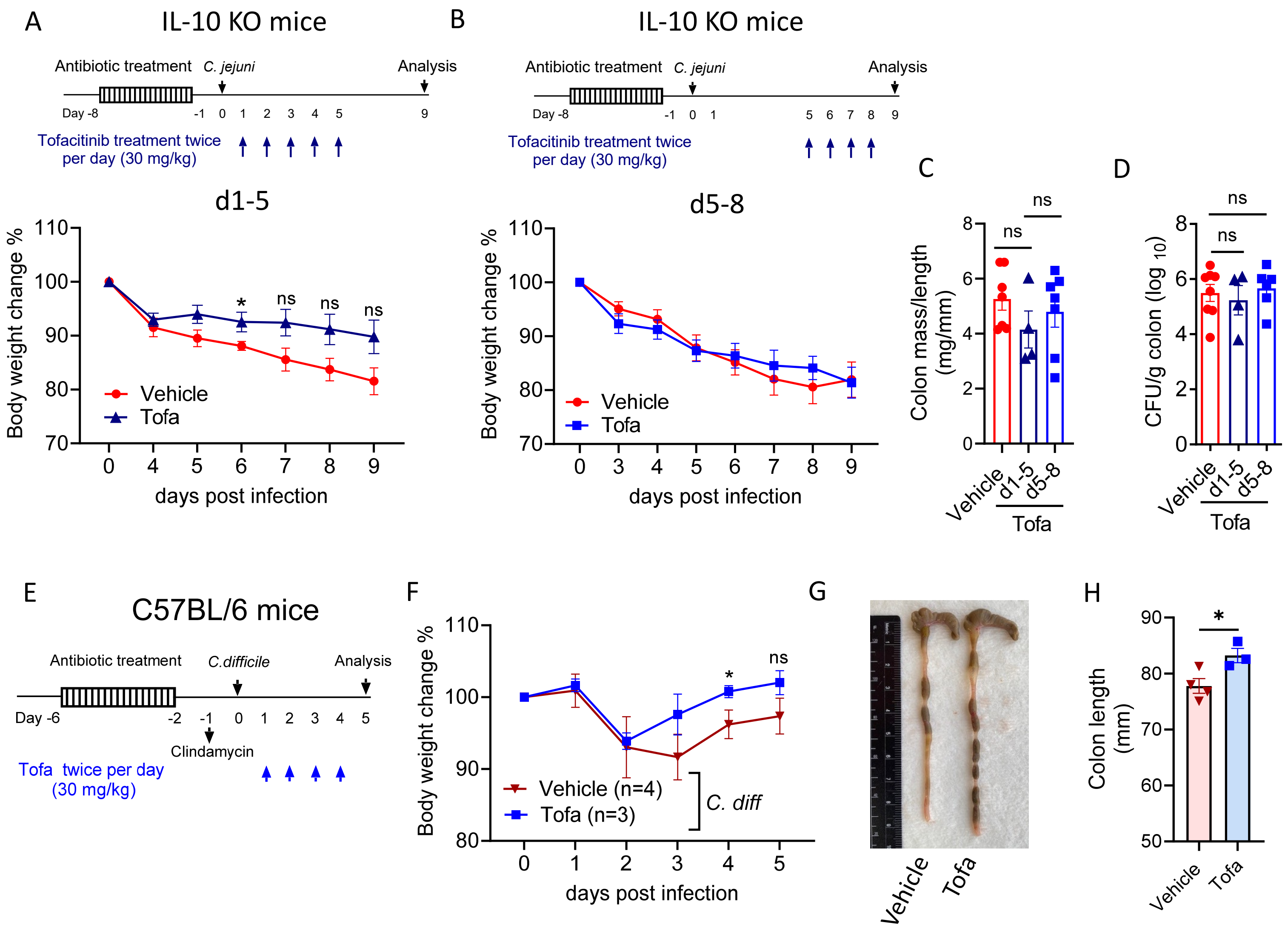
A
IL-10 KO mice
Antibiotic treatment
C. jejuni
Analysis
Day -8
-1 0 1 2 3 4 5 9
Tofacitinib treatment twice per day (30 mg/kg)
d1-5
Body weight change %
days post infection
Vehicle
Tofa
B
IL-10 KO mice
Antibiotic treatment
C. jejuni
Analysis
Day -8
-1 0 1 5 6 7 8 9
Tofacitinib treatment twice per day (30 mg/kg)
d5-8
Body weight change %
days post infection
Vehicle
Tofa
C
Colon mass/length (mg/mm)
Vehicle
d1-5
d5-8
Tofa
D
CFU/g colon (log 10)
Vehicle
d1-5
d5-8
Tofa
E
C57BL/6 mice
Antibiotic treatment
C.difficile
Analysis
Day -6
-2 -1 0 1 2 3 4 5
Clindamycin
Tofa twice per day (30 mg/kg)
F
Body weight change %
Vehicle (n=4)
Tofa (n=3)
C. diff
days post infection
G
Vehicle
Tofa
H
Colon length (mm)
Vehicle
Tofa

Fig S2

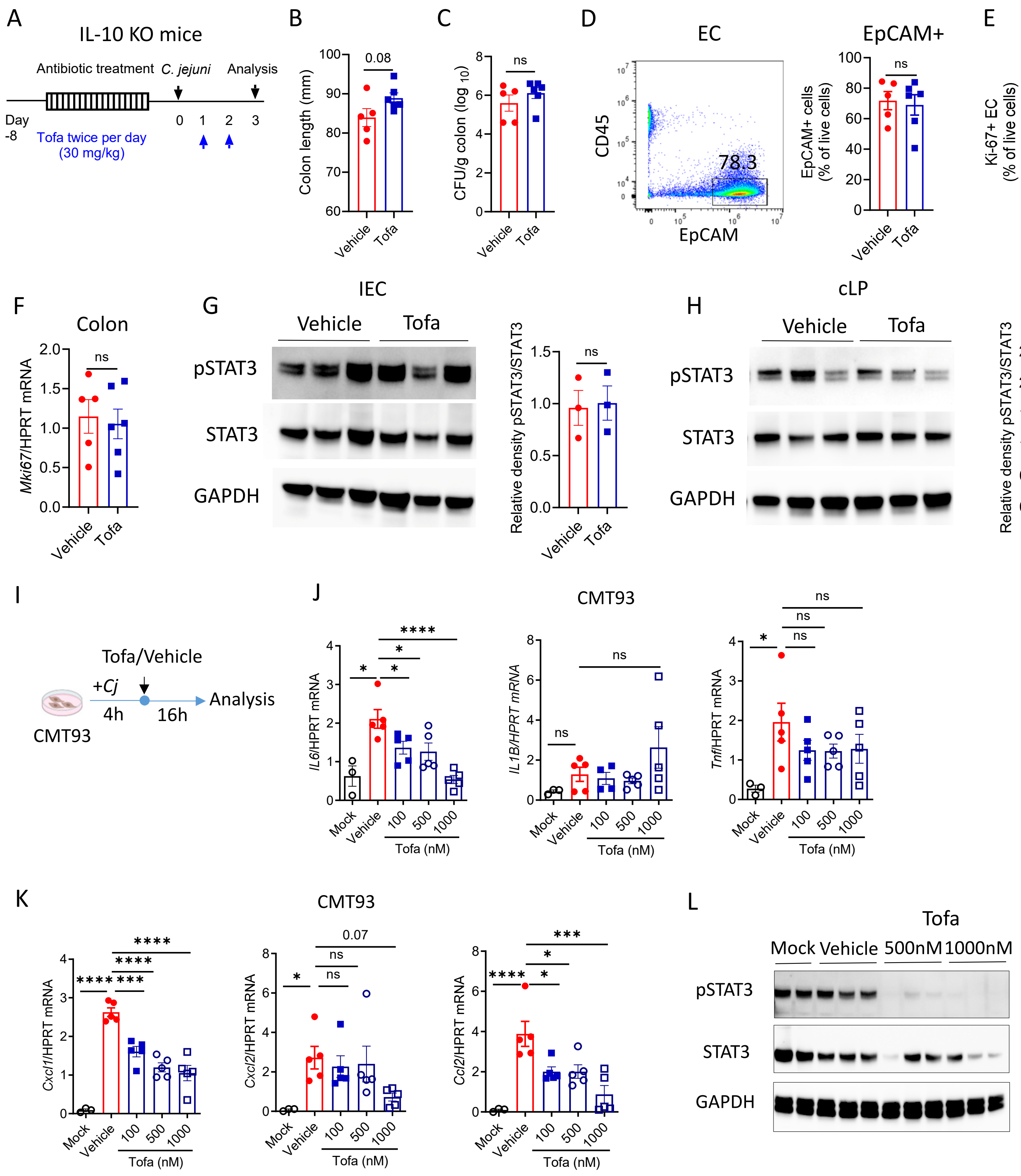

A
IL-10 KO mice
Antibiotic treatment
C. jejuni
Analysis
Day
-8
0
1
2
3
Tofa twice per day
(30 mg/kg)
B
Colon length (mm)
0.08
Vehicle
Tofa
C
CFU/g colon (log 10)
ns
D
EC
CD45
EpCAM
78.3
EpCAM+
EpCAM+ cells
(% of live cells)
E
Ki-67+
Ki-67+ EC
(% of live cells)
F
Colon
Mki67/HPRT mRNA
G
IEC
pSTAT3
STAT3
GAPDH
Relative density pSTAT3/STAT3
H
cLP
I
Tofa/Vehicle
+Cj
4h
16h
Analysis
CMT93
J
IL6/HPRT mRNA
IL1B/HPRT mRNA
Tnf/HPRT mRNA
Mock
100
500
1000
Tofa (nM)
K
Cxcl1/HPRT mRNA
Cxcl2/HPRT mRNA
Ccl2/HPRT mRNA
0.07
L
Tofa
Mock
Vehicle
500nM
1000nM

Fig S3

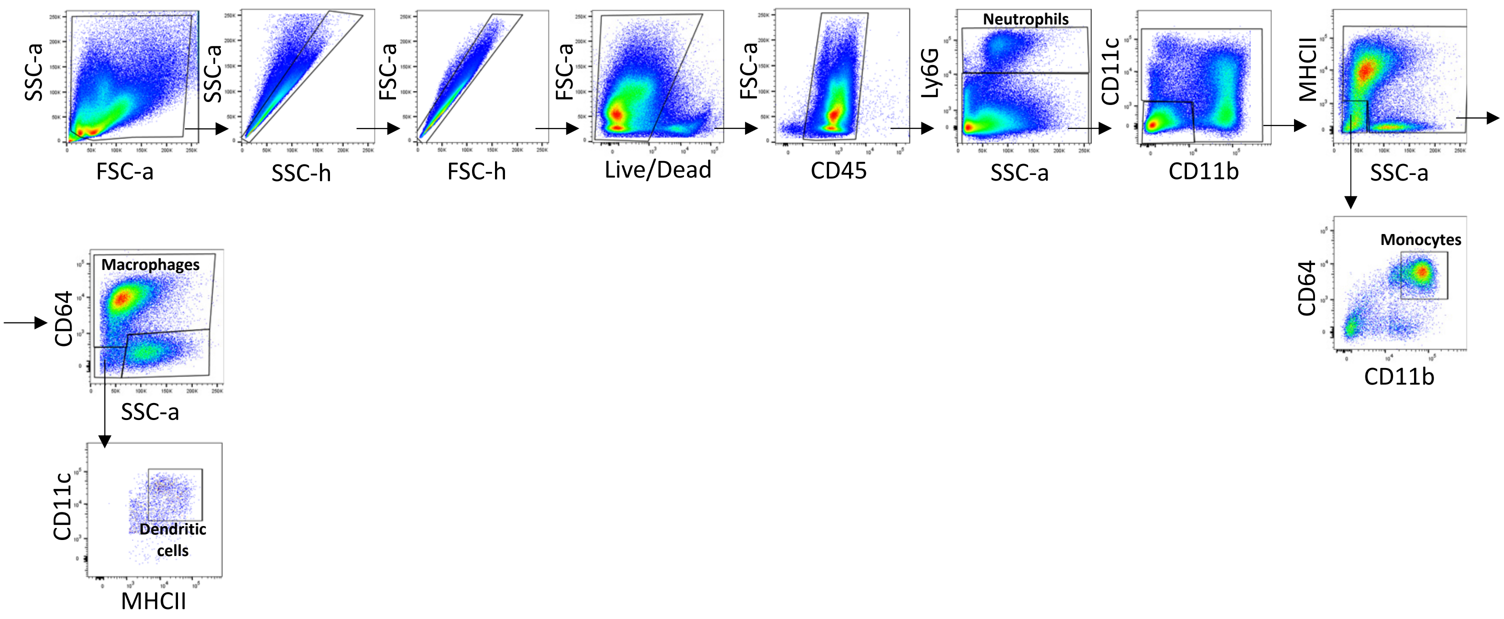
SSC-a
FSC-a
SSC-a
SSC-h
FSC-a
FSC-h
FSC-a
Live/Dead
FSC-a
CD45
Neutrophils
Ly6G
SSC-a
CD11c
CD11b
MHCII
SSC-a
Monocytes
CD64
CD11b
Macrophages
CD64
SSC-a
CD11c
Dendritic cells
MHCII

Fig S4

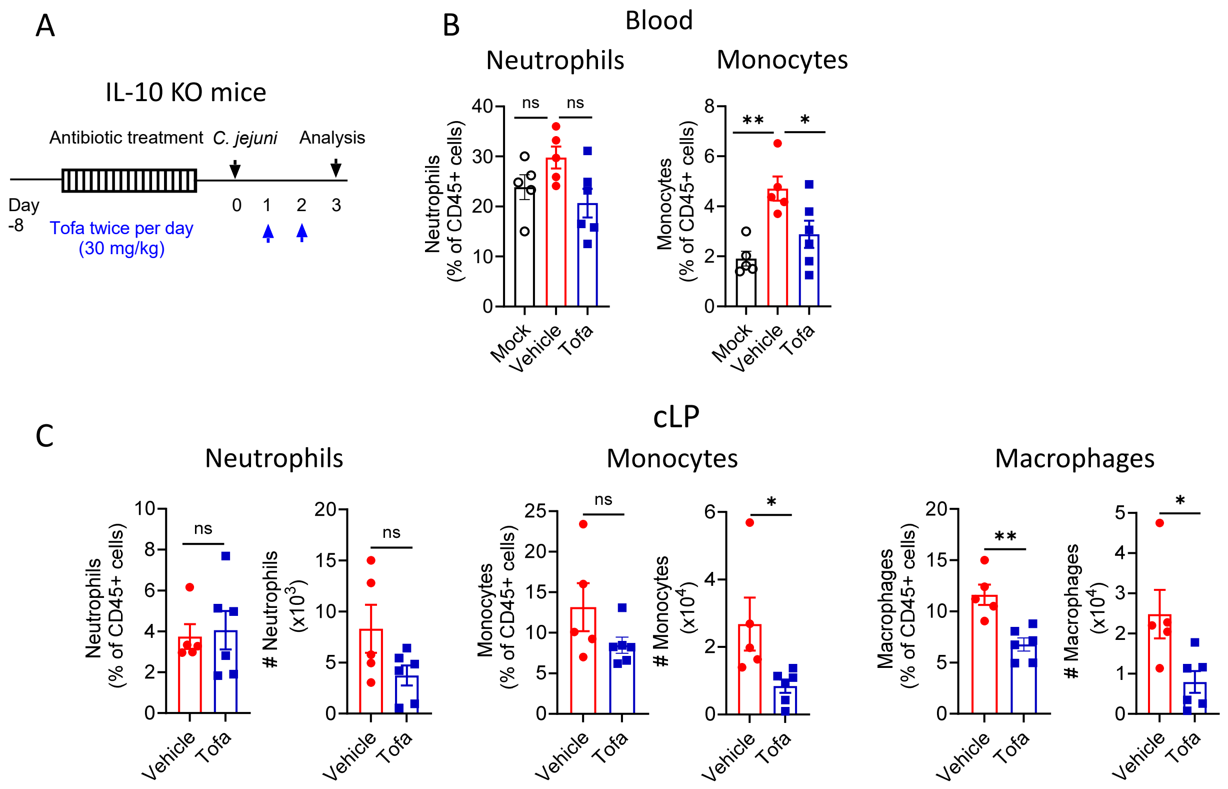
A
IL-10 KO mice
Antibiotic treatment
C. jejuni
Analysis
Day
-8
0
1
2
3
Tofa twice per day
(30 mg/kg)
B
Blood
Neutrophils
Monocytes
Neutrophils
(% of CD45+ cells)
Monocytes
(% of CD45+ cells)
ns
*
**
Mock
Vehicle
Tofa
C
cLP
Neutrophils
Monocytes
Macrophages
Neutrophils
(x10^3)
Monocytes
(x10^4)
Macrophages
(% of CD45+ cells)
Macrophages
(x10^4)

Fig S5

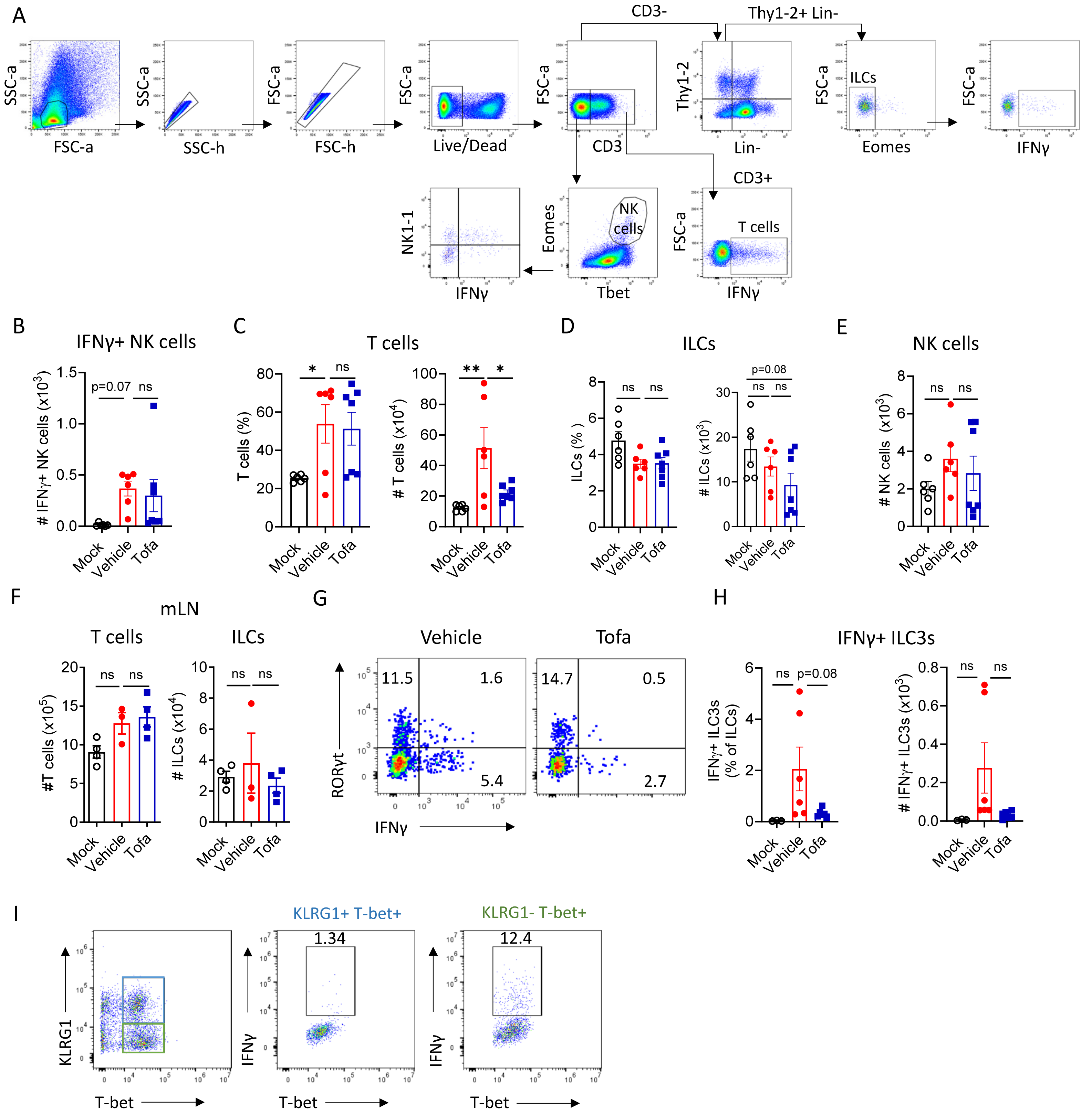
A
CD3-
Thy1-2+ Lin-
SSC-a
FSC-a
SSC-h
FSC-h
Live/Dead
CD3
Thy1-2
Lin-
ILCs
Eomes
IFNγ
CD3+
NK1-1
NK cells
Tbet
T cells
B
IFNγ+ NK cells
# IFNγ+ NK cells (x10^3)
p=0.07
ns
C
T cells
T cells (%)
T cells (x10^4)
*
**
D
ILCs
ILCs (%)
# ILCs (x10^3)
p=0.08
E
NK cells
NK cells (x10^3)
Mock
Vehicle
Tofa
F
mLN
#T cells (x10^5)
ILCs (x10^4)
G
Vehicle
Tofa
RORγt
11.5
1.6
5.4
14.7
0.5
2.7
H
IFNγ+ ILC3s
IFNγ+ ILC3s (% of ILCs)
IFNγ+ ILC3s (x10^3)
I
KLRG1+ T-bet+
KLRG1- T-bet+
KLRG1
T-bet
1.34
12.4

Fig S6

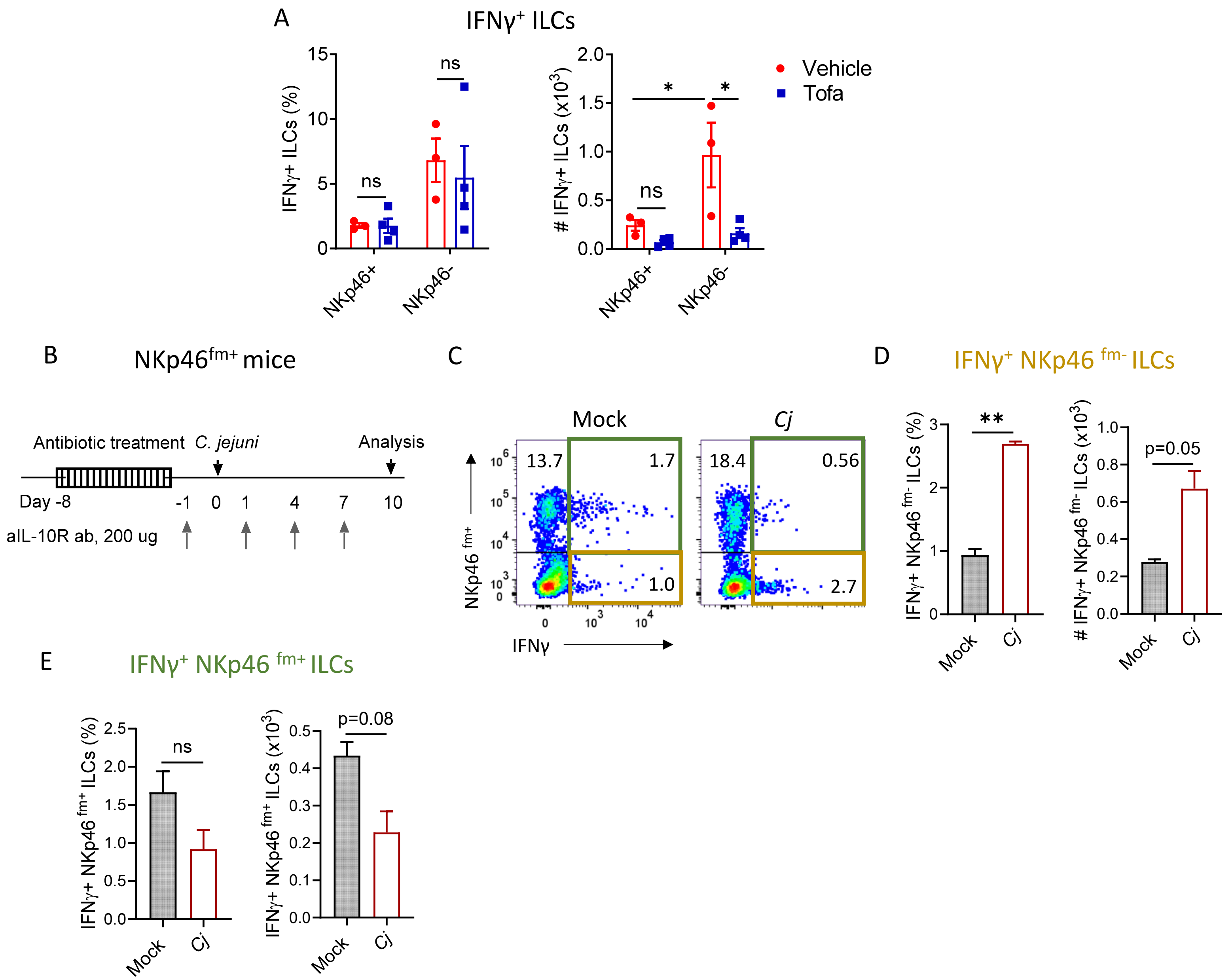

A
IFNγ+ ILCs
IFNγ+ ILCs (%)
IFNγ+ ILCs (x10^3)
ns
*
Vehicle
Tofa
NKp46+
NKp46-
B
NKp46fm+ mice
Antibiotic treatment
C. jejuni
Analysis
Day -8
-1
0
1
4
7
10
aIL-10R ab, 200 ug
C
Mock
Cj
13.7
1.7
1.0
18.4
0.56
2.7
NKp46 fm+
IFNγ
D
IFNγ+ NKp46 fm- ILCs
IFNγ+ NKp46 fm- ILCs (%)
IFNγ+ NKp46 fm- ILCs (x10^3)
**
p=0.05
E
IFNγ+ NKp46 fm+ ILCs
IFNγ+ NKp46 fm+ ILCs (%)
IFNγ+ NKp46 fm+ ILCs (x10^3)
p=0.08

Fig S7

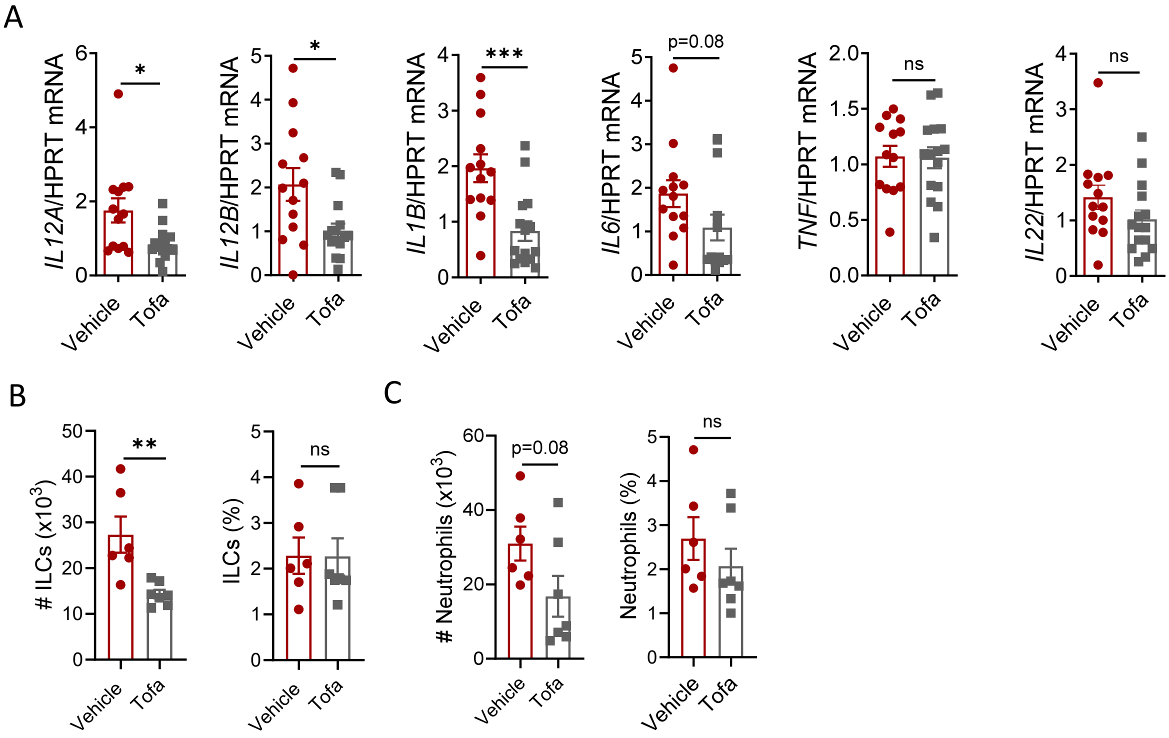
A
IL12A/HPRT mRNA
IL12B/HPRT mRNA
IL1B/HPRT mRNA
IL6/HPRT mRNA
TNF/HPRT mRNA
IL22/HPRT mRNA
*
***
p=0.08
ns
Vehicle
Tofa
B
ILCs (x10^3)
ILCs (%)
**
C
Neutrophils (x10^3)
Neutrophils (%)

Fig S8

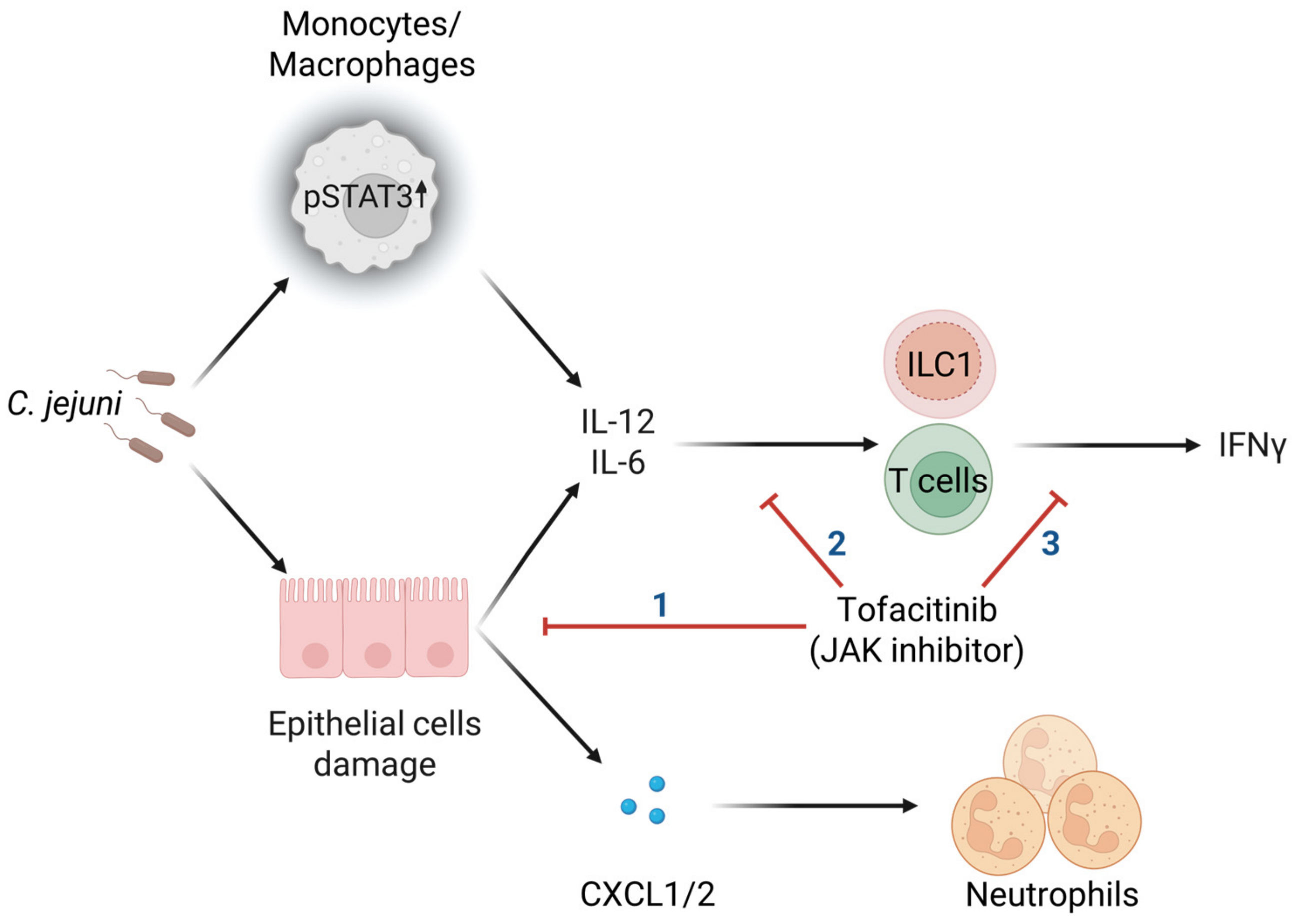
Monocytes/
Macrophages
pSTAT3↑
C. jejuni
IL-12
IL-6
ILC1
T cells
IFNγ
2
3
1
Tofacitinib
(JAK inhibitor)
Epithelial cells
damage
CXCL1/2
Neutrophils
